# Niche-Associated Activation of Rac Promotes the Asymmetric Division of *Drosophila* Female Germline Stem Cells

**DOI:** 10.1371/journal.pbio.1001357

**Published:** 2012-07-03

**Authors:** Wen Lu, M. Olivia Casanueva, Anthony P. Mahowald, Mihoko Kato, David Lauterbach, Edwin L. Ferguson

**Affiliations:** 1 Committee on Genetics, Genomics and Systems Biology, University of Chicago, Chicago, Illinois, United States of America; 2 Committee on Development, Regeneration and Stem Cell Biology, University of Chicago, Chicago, Illinois, United States of America; 3 Department of Molecular Genetics and Cell Biology, University of Chicago, Chicago, Illinois, United States of America; Johns Hopkins University Medical School, United States of America

## Abstract

**Background:**

*Drosophila* female germline stem cells (GSCs) reside adjacent to a cellular niche that secretes Bone Morphogenetic Protein (BMP) ligands and anchors the GSCs through adherens junctions. The GSCs divide asymmetrically such that one daughter remains in the niche as a GSC, while the other is born away from the niche and differentiates. However, given that the BMP signal can be diffusible, it remains unclear how a local extracellular asymmetry is sufficient to result in a robust pattern of asymmetric division.

**Methods and Findings:**

Here we show that GSCs are polarized with respect to the cellular niche. We first use a modified biosensor to demonstrate that the small GTPase Rac is asymmetrically activated within the GSC at the niche-GSC interface. Experiments using loss-of-function and gain-of-function mutations in Rac indicate that asymmetric Rac activity both localizes the microtubule binding protein Apc2 to orient one GSC centrosome at the niche-GSC interface during interphase and activates the Jun N-terminal kinase pathway to increase the ability of the GSC to respond to BMP ligands. Other processes act in concert with each function of Rac. Specifically, we demonstrate that the GSC cell cycle arrests at prometaphase if centrosomes are misoriented.

**Conclusions:**

Thus, the GSCs, an adult stem cell present in a cellular niche, have a niche-associated polarity that couples control of the division plane with increased response to an extracellular maintenance signal. Other processes work in parallel with the Rac-mediated polarity to ensure a robust pattern of asymmetric division. We suggest that all adult stem cells likely employ multiple, independently acting mechanisms to ensure asymmetric division to maintain tissue homeostasis.

## Abstract 2

An intracellular polarity in activation of the Rac GTPase cooperates with an extracellular signal to ensure a robust pattern of stem cell division in *Drosophila* female germline stem cells.

## Author Summary

Adult stem cells are undifferentiated cells within an organism that undergo continual asymmetric division to produce two daughter cells that have different cell fates: one daughter remains a stem cell like its parent, while the other daughter differentiates. Thus the stock of stem cells is renewed while new cells are provided to ensure tissue maintenance. Often, an adult stem cell is present within a niche, a specific microenvironment that contains signals necessary to maintain the stem cell in an undifferentiated state. The plane of division of a niche-resident stem cell can be controlled so that one of its daughters arises outside of the niche. This daughter does not receive the maintenance signals and differentiates. However, because biological systems are inherently noisy, we postulated that there might be mechanisms that couple the control of the plane of stem cell division to the response to the extracellular maintenance signals to ensure a continued pattern of asymmetric division. In this study, we show that a well-characterized niche-resident stem cell, the *Drosophila* female germline stem cell (GSC), also has an intracellular polarity that contributes to the determination of daughter cell fate. We find that the small GTPase Rac is activated at the interface between the GSC and its niche and that this localized Rac activity has two functions: first, it orients the plane of GSC division to ensure that one GSC daughter is born outside the niche; and second, it increases the ability of the niche-resident GSC to respond to the maintenance signal. We propose that most stem cells integrate multiple mechanisms to ensure a robust pattern of asymmetric division.

## Introduction

A robust pattern of asymmetric cell division underlies the ability of adult stem cells to balance self-renewal and differentiation to ensure tissue homeostasis. In principle, either of two mechanisms could underlie the asymmetric cell division: first, a polarization in the stem cell could result in the asymmetric segregation of cytoplasmic determinants to one daughter; alternatively, the two descendant sister cells could be initially equivalent but respond differently to specific stimuli in their microenvironments [1]. However, in either case, the mechanism that generates the asymmetry must be coordinated with the control of the plane of cell division. For example, in *Drosophila* neuroblasts, a polarity within the stem cell, mediated by the asymmetric localization of multiple protein complexes that are organized by the Par-3 homolog Bazooka, couples the asymmetric segregation of cellular determinants with control of the orientation of the neuroblast division plane [2]. In contrast, a cellular niche could promote an asymmetric self renewal division in an adult stem cell by secreting a locally acting maintenance factor and by orienting the plane of stem cell division such that one daughter is born outside the niche and is not exposed to the maintenance factor [3]. However, it remains an open question whether a stem cell residing in a cellular niche could also have a polarity that links the response to the maintenance signal with a mechanism that orients the division plane.

**Figure 1:**
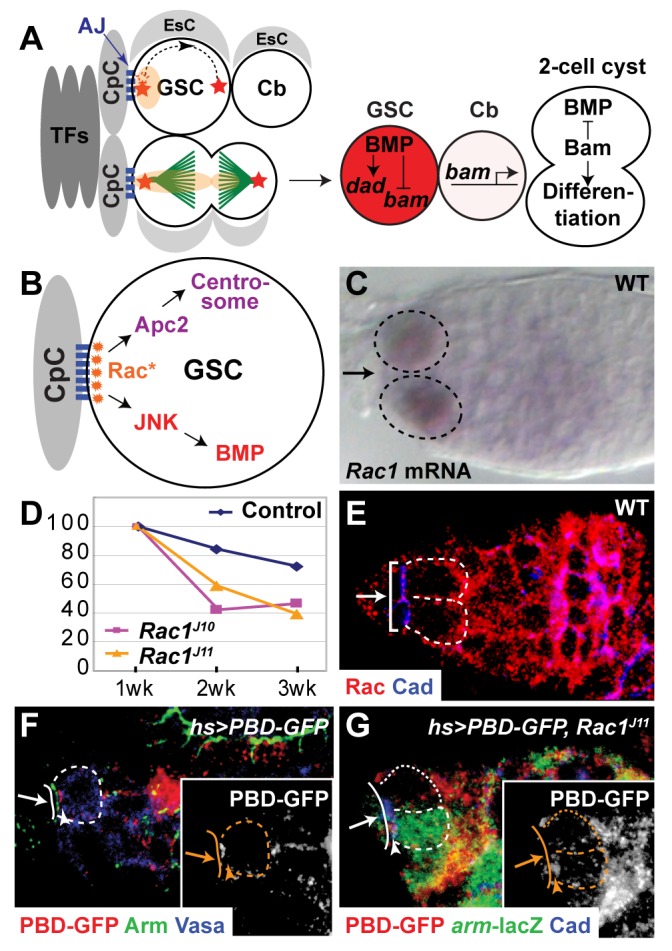
Activated *Rac* is localized to the CpC-GSC interface. (A) GSCs in the CpC niche. Two to three GSCs are present in a niche composed of Terminal Filament cells (TFs), Cap Cells (CpCs), and Escort Cells (EsCs). The GSCs are anchored to the CpCs by Adherens Junctions (AJs). Each GSC contains a cytoskeletal organelle, the spectrosome (light orange) adjacent to the niche. One centrosome (red star) is present at the niche-GSC interface. After centrosome duplication, a centrosome migrates around the cortex (dotted line with arrow). The plane of GSC division is perpendicular to the niche-GSC interface. One daughter cell, the Cystoblast (Cb), is born outside of the niche and the other daughter cell remains in the niche as a GSC. During GSC division, the spectrosome elongates, and some spectrosomal material is segregated to the Cb. GSCs have high levels of BMP signaling, which leads to the transcription of *dad* and the repression of *bam* expression. Cbs have much lower levels of BMP signaling and transcribe *bam*. Bam functions to promote Cb differentiation and to inhibit BMP signaling. (B) Model for Rac function in GSCs: Rac is asymmetrically activated (Rac*) at the CpC-GSC interface to orient interphase centrosomes and to promote BMP signaling in GSCs. (C) Wild-type ovariole in situ hybridized with *Rac1* anti-sense probe (purple). Some, but not all, GSCs expressed *Rac* transcript. (D) Percentage of germaria carrying marked wild-type or *Rac* mutant clones as a function of time. Wild type (blue diamond), *Rac1^J10^ Rac2^Δ^ Mtl^Δ^*/*Rac1^J11^ Rac2^Δ^*+ (purple rectangle), and *Rac1^J11^ Rac2^Δ^ Mtl^Δ^*/*Rac1^J11^ Rac2^Δ^*+ (yellow triangle). (E) Rac is localized to the cortex of wild-type GSCs. Bracket, the CpC-GSC interface as marked by anti-Cadherin staining. (F) After mild heat shock, a biosensor for activated Rac, PBD-GFP, is localized at the CpC-GSC interface in wild-type GSCs. (G) PBD-GFP is not localized to the CpC-GSC interface after mild-heat shock in a *Rac* mutant (*Rac1^J11^ Rac2^Δ^ Mtl^Δ^*/*Rac1^J11^ Rac2^Δ^+*) GSC (dotted outline), marked by the absence of *lacZ* expression, compared to normal localization at the CpC-GSC interface in a *Rac1^J11^ Rac2^Δ^ Mtl^Δ^*/+++ GSC (dashed outline). (C, E–G) Arrow, CpC niche; dashed or dotted outline, individual GSC. (F–G) Solid line, CpC-GSC interface; arrowhead, asymmetrically localized PBD-GFP in GSCs. Inset: white, anti-GFP staining.

Both female and male germ line stem cells (GSCs) in *Drosophila* are present in well-defined cellular niches [4]. In females, the niche comprises three somatic cell types: the terminal filament cells, which are the most-anterior cells in each ovariole; a small number of the Cap Cells (CpCs), which form adherens junctions with the GSCs; and escort cells, which enwrap each GSC with thin cytoplasmic extensions (Figure 1A) [5]. The female niche is of limited size and can accommodate only two to three GSCs. CpCs secrete BMP ligands that are necessary and sufficient for GSC maintenance [6],[7]. Each GSC, which contains a cytoskeletal rich organelle called the spectrosome adjacent to the niche, divides with an invariant mitotic spindle orientation perpendicular to the niche-GSC interface [8]. The daughter born in the niche has high levels of BMP signaling and remains a GSC, while the daughter born outside the niche has much lower levels of BMP signaling and becomes a Cystoblast (Cb) [9]–[11]. The reduction in BMP signaling in Cbs allows expression of *bag of marbles* (*bam*) [11],[12], which promotes Cb differentiation by transiently downregulating two translational inhibitors, Nanos and Pumilio [13],[14], and by blocking BMP signal transduction downstream of the BMP receptors [10]. The Cbs undergo four rounds of synchronized division with incomplete cytokinesis to generate a cyst composed of 16 cells connected by a branched fusome derived from the spectrosome. One cyst cell becomes an oocyte; the others become polyploid nurse cells [15].

The structure of the male niche at the tip of the testis is similar to, but different from, the female niche. The male niche comprises two somatic cell types, the hub cells, which cluster together to form a cellular cone surrounded by a circle of 6–12 GSCs, and the cyst stem cells [4]. Similar to female GSCs, male GSCs are anchored by adherens junctions to the hub cells, and divide with an orientation that is perpendicular to the niche-GSC interface [16]. The GSC daughter born away from the niche differentiates into a Gonialblast that divides four times to produce 16 interconnected spermatogonia, each of which undergoes meiosis to give rise to four sperm. The hub cells secrete Unpaired, a ligand for the JAK/STAT pathway, which is necessary for continued GSC maintenance [17]. Recent results suggest Upd is necessary for GSC adhesion to the niche and likely acts upon the cyst stem cells to create a protected microenvironment for the GSCs [18], possibly including secretion of a BMP ligand [19].

In both sexes, the GSCs divide with an orientation perpendicular to the niche-GSC interface. In male GSCs, adherens junctions between the hub cells and the GSC localize the astral microtubule binding protein Apc2 at the niche-GSC interface to position the GSC centrosome during interphase [16],[20],[21]. After centrosome duplication, the mother centrosome, which has a robust array of microtubules, remains at the niche-GSC interface, while the daughter centrosome, which has few associated microtubules, migrates around the cortex to orient the mitotic spindle perpendicular to the niche-GSC interface [16],[20]. Moreover, misorientation of the centrosomes results in a cell cycle arrest after centrosome duplication in the G2 stage [22]. Thus, in males, the adherens junctions provide a polarity cue that orients an interphase centrosome to presage the orientation of the mitotic spindle and the plane of GSC division, but a second mechanism, a G2 cell cycle arrest, acts to ensure the robustness of the division plane.

Although the mitotic plane of female GSCs is also oriented perpendicular to the niche-GSC interface, the mechanism by which it is done is less clear. In females, unlike males, the spectrosome abuts the GSC-niche interface, and *hts* mutants, which lack a morphologically identifiable spectrosome, have a randomized orientation of the mitotic spindle [8], suggesting that the spectrosome may aid in the positioning of the spindle.

In both male and female GSCs, a change in orientation of the mitotic spindle is correlated with a change in daughter cell fate. Randomization of male GSC centrosome orientation, by mutations in *Apc2* or the centrosomal component *centrosomin*, causes an increase in GSC number [16], as a larger number of GSC daughters become associated with the niche. Likewise, if one female GSC is lost from the niche, the remaining GSC divides with a spindle oriented parallel to the niche-GSC interface such that both daughters remain in the niche and adopt GSC fates [7]. These observations suggest that there may be a mechanism that directly couples the orientation of the plane of GSC division to the response to the extracellular maintenance signal.

In this article, we show that in female GSCs the small GTPase Rac is asymmetrically activated at the GSC-niche interface. Activated Rac both orients interphase centrosomes by localizing Apc2 to the niche-GSC interface and increases GSC responsiveness to the BMP signal by activating the Jun N-terminal kinase (JNK) pathway (Figure 1B). Both actions of Rac are functionally redundant with other processes, likely to ensure robustness of this asymmetric division. Thus, in GSCs, a niche-associated polarity influences both the plane of cell division and the response to the extracellular maintenance signal.

## Results

### Rac Is Asymmetrically Activated in GSCs

*Rac1* was identified in a microarray experiment for genes that are expressed in GSCs (see Text S1). In situ hybridization validated *Rac1* transcription in some wild-type GSCs (Figure 1C) and in some GSC-like cells in tumorous germaria caused by expression of a constitutively active form of the BMP type I receptor Thickveins (*TkvAct*) [10] (Figure S1A). Because *Rac1* is not expressed in all GSCs at all times, *Rac1* transcription is likely modulated by other factors, possibly including the state of the cell cycle. The three Rac family members in *Drosophila*, *Rac1*, *Rac2*, and *Mtl* have partially redundant functions in other tissues [23]. Although in situ hybridization failed to reveal germline transcription of *Rac2* or *Mtl* (unpublished data), we investigated the function of the Rac GTPases in GSC maintenance by eliminating the activity of as many family members as possible. Thus, we used mitotic recombination to generate clones of GSCs homozygous for *Rac1* and *Rac2* mutations and heterozygous for an *Mtl* mutation (Figure S1B), referred to as “*Rac* mutant GSCs.” We compared the persistence of *Rac* mutant GSC clones to control wild-type GSC clones in the anterior germarium. While the half-life of wild-type GSC clones was over 4 wk, GSC clones mutant for *Rac2* and either the hypomorphic allele *Rac1^J10^* or the null allele *Rac1^J11^* had a half-life of 2 wk (Figure 1D), demonstrating that Rac promotes GSC maintenance. However, *Rac* mutant GSCs are lost more slowly than GSCs defective in BMP signal transduction [6] or GSCs defective in adherens junctions [5], both of which have a half-life of approximately 3–5 d.

An anti-human Rac1 antibody stained the cortex of all germ cells including the niche-GSC interface, which is marked by the adherens junction component, DE-Cadherin (Figure 1E and Figure S1C). Anti-Rac staining was increased in germaria in which a constitutively active allele of *Rac1*, *Rac1^V12^*, was expressed using a germline-specific driver, *nos*-Gal4 (Figure S1D), and markedly reduced in germaria in which RNAi constructs against *Rac1* and *Rac2* were expressed using the *nos*-Gal4 driver (Figure S1E), referred to as “*Rac-RNAi* GSCs,” confirming the antibody specifically recognizes *Drosophila* Rac proteins.

To determine the subcellular localization of active GTP-bound Rac in GSCs, we adapted a previously reported biosensor, the P21-binding-domain (PBD) of P21-activated Kinase that only binds to activated Rac and Cdc42 [24]. We used a mild heat-shock regimen to transiently express a chimeric protein composed of the *Drosophila* PBD fused to GFP (Green Fluorescent Protein) and assayed its subcellular localization 3 h after heat shock. Although there was variability in expression levels after heat shock (see Text S1), in all interphase GSCs with asymmetric GFP staining, PBD-GFP staining was present at the niche-GSC interface (Figure 1F; Figures S1F–H). The ratio of intensity of staining per unit area at the niche-GSC interface to the remainder of GSC cytoplasm was 90∶1 (91±25∶1, *n* = 10, Figure S1I). Thus, in GSCs, Rac is asymmetrically activated at the niche-GSC interface.

To confirm the specificity of the assay, we demonstrated that transient expression of GFP alone resulted in staining throughout GSCs (Figure S1J), that co-expression of active Rac1^V12^ protein and PBD-GFP also caused staining throughout GSCs (Figure S1K), and that PBD-GFP failed to localize to the niche-GSC interface in *Rac* mutant GSCs (Figure 1G). In addition, PBD-GFP localized properly in GSCs mutant for a partial-loss-of function *cdc42* allelic combination (Figure S1L).

### Rac Acts through Apc2 to Orient GSC Centrosomes

To begin to characterize Rac function in GSCs, we examined whether loss or gain of *Rac* activity affected orientation of the GSC division plane. Because orientation of the centrosome at the niche-GSC interface predicts the plane of division in male GSCs, we first examined centrosome orientation in wild-type female GSCs in both fixed and living specimens. Overall, 96% of GSCs (*n* = 92, 72 fixed+20 living) had one centrosome associated with the niche-GSC interface.

**Figure 2:**
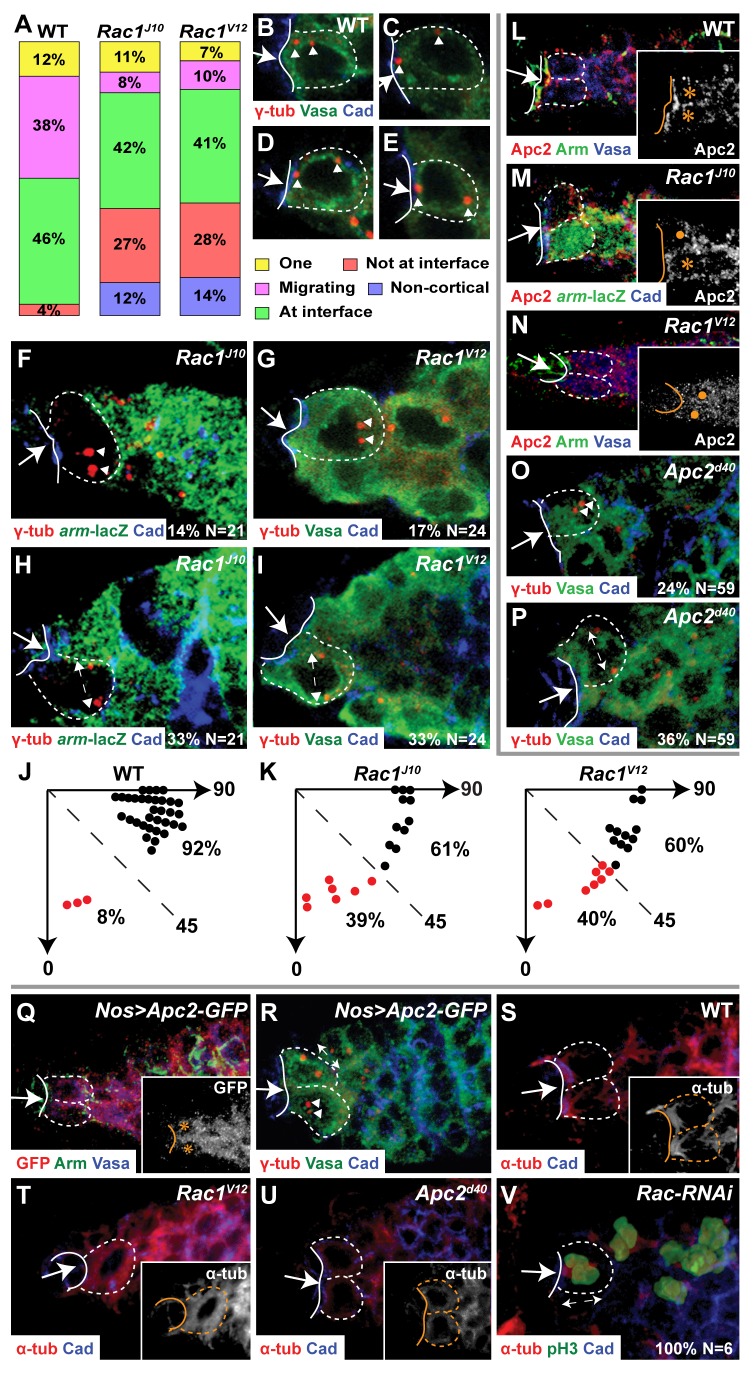
Asymmetric Rac activity localizes Apc2 to control centrosome position in interphase GSCs. (A) Bar graphs of GSC centrosome phenotypes in wild-type, *Rac* mutant, or *Rac^V12^* GSCs. One, one cortical centrosome located at the CpC-GSC interface; Migrating, two cortical centrosomes one at the interface and one migrating; At interface, two cortical centrosomes separated by 180° one of which is at the interface; Not at interface, two cortical centrosome separated by 180° neither of which is at the interface; Non-cortical, one or both centrosomes detached from cortex. (B–E) Cortical migration of one centrosome after centrosome duplication in wild-type GSCs (arrowhead, centrosome). (F–I) Defects in centrosome position in *Rac* mutant (*Rac1^J10^ Rac2^Δ^ Mtl^Δ^*/*Rac1^J10^ Rac2^Δ^+*) (F,H) or in *Rac1^V12^* GSCs (G,I). (F,G) At least one centrosome is not at cortex (arrowheads). (H,I) In GSCs with two cortical centrosomes separated by 180° (double-headed arrows), neither centrosome is at CpC-GSC interface. (J–K) Graphs with angles, in individual control GSCs (J) or GSCs with perturbations in *Rac* activity (K), between the CpC-GSC interface and the line connecting two cortical centrosomes separated by 180°. Black dot, one of two centrosomes at the CpC-GSC interface. Red dot, neither centrosome at the CpC-GSC interface. Percentage (%) of GSCs with each phenotype compared to total in the graph. (L–N) Apc2 concentration at the CpC-GSC interface (L) is disrupted in GSCs with altered Rac activity (M,N). Inset: white, anti-Apc2 staining; asterisk, control GSC (L,M); dot, *Rac* mutant GSC (M) or *Rac1^V12^* GSC (N). (O–P) *Apc2^d40^* GSC with non-cortical centrosomes (O, arrowheads); *Apc2^d40^* GSC with two cortical centrosomes, neither of which is at the CpC-GSC interface (P, double-headed arrow). (Q) Uniform cytoplasmic localization of Apc2-GFP expressed in the germ line. Inset: white, anti-GFP; asterisk, Apc2-GFP-expressing GSC. (R) Expression of Apc2-GFP causes the same classes of centrosome defects as are observed in *Apc2^d40^* mutants: a GSC with two cortically located centrosomes, neither of which is present at the CpC-GSC interface (double-headed arrow), and a GSC with centrosomes detached from the cortex (arrowheads). (S) In wild-type interphase GSCs, microtubules are organized as a bundled network near the CpC-GSC interface. (T) In *Rac1^V12^*-expressing GSCs, a microtubule network is present uniformly around the GSC cell cortex. (U) In *Apc2^d40^* GSCs, the microtubule staining is reduced and uniformly localized. (S–U) Inset: white, anti-α-tubulin staining. (V) A *Rac-RNAi* GSC with a mitotic spindle (double-headed arrow) perpendicular to the CpC-GSC interface and with one pole at the interface. All mitotic *Rac-RNAi* GSCs have the same orientation of the mitotic spindle. (B–I,O–P,R) Anti-γ-tubulin, centrosomes. (B–E,G,I,L,N–R) Anti-Vasa, germline cytoplasm. (F,H,M) Absence of anti-β-galactosidase, *Rac* mutant GSC. (S–V) Anti-α-tubulin, cytoplasmic microtubules (S–U), and mitotic spindle (V). (B–I,L–V) Arrow, CpC niche; solid line, CpC-GSC interface; dashed outline, individual GSC. (F–I,O–P,V) Percentage (%) of total (*N*) GSCs with the specific phenotype displayed in the panel.

In fixed specimens (Figure 2A), 12% of GSCs were at a stage in the cell cycle prior to centrosome duplication. In all these GSCs, the single centrosome was cortically located at the niche-GSC interface. The remaining GSCs had two centrosomes. In 38% of GSCs, one centrosome was associated with the niche-GSC interface while the other centrosome was migrating around the GSC cortex (Figure 2B–D). In 46% of GSCs, one centrosome was associated with the interface, and the two centrosomes were separated by 180°, forming an angle of 45°–90° with the niche-GSC interface (Figure 2E). In the remaining 4% of GSCs, neither centrosome was at the interface, and the centrosome pair was oriented parallel to the interface, suggesting these GSCs could have been preparing to undergo a symmetric self-renewal division [7]. Thus, 92% of wild-type GSCs with two centrosomes separated by 180° had one centrosome at the niche-GSC interface (Figure 2J).

We also recorded centrosome behavior in living GSCs using a GFP-tagged Centrosomin (*n* = 20). While all GSCs had a niche-associated centrosome, in 35% of GSCs, one centrosome was migrating around the GSC cortex (Movie S1 and Figure S2), and in 35% of GSCs, the two centrosomes were separated by 180° (Movie S2). Taken together, our data indicate that, unlike a previous report [25], centrosomes in female GSCs are positioned similarly to those in male GSCs [16].

Quantitation of GSC centrosomes in fixed *Rac* mutant GSCs or in GSCs expressing *Rac1^V12^* (Figure 2A) indicated two patterns of centrosome orientation that were not present at high levels in wild-type GSCs. First, in 12%–14% of GSCs with perturbations in *Rac* activity, one or both centrosomes were not located at the GSC cortex (Figure 2F and 2G). Second, 27%–28% of GSCs had two cortical centrosomes separated by 180°, neither of which was associated with the niche-GSC interface (Figure 2H and 2I). Quantitation of the angle of the centrosome pair in GSCs with two centrosomes separated by 180° indicated that centrosomes in 40% of GSCs of both genotypes were not associated with the niche and formed an angle of 0°–45° with the niche-GSC interface (Figure 2K). Because complete randomization of orientation of GSC centrosome pairs would result in 50% of GSCs in which this angle was between 0° and 45°, we conclude that both loss and gain of *Rac* function essentially randomize GSC centrosome orientation. Asymmetric Rac activity is thus strongly correlated with the localization of one centrosome to the niche-GSC interface.

We then examined whether Rac affects centrosome localization in male GSCs. In 2-d-old wild-type testes, 97% (*n* = 73) of GSCs had a centrosome associated with the niche-GSC interface (Figure S3A). In contrast, in 2-d-old testes, 28% (*n* = 83) of *Rac* RNAi GSCs and 46% (*n* = 95) of *Rac^V12^* GSCs did not have a centrosome associated with the niche-GSC interface (Figure S3B and S3C). In males, mutations that alter centrosome orientation can cause an increase in GSC number, as a result of an altered plane of GSC division [16]. Two-day-old wild-type testes had an average of 8.9±0.8 (*n* = 18) GSCs, while 2-d-old testes with *Rac* RNAi GSCs had an average of 10.4±1.5 GSCs (*n* = 8, *p* = 0.03, Welch two sample *t*-test) and 2-d-old testes with *Rac^V12^* GSCs had an average of 11.9±1.7 GSCs (*n* = 8, *p* = 0.001). Thus, alterations in *Rac* activity lead to misorientation of male GSC centrosomes and result in an increase in male GSC number.

In male GSCs, Apc2 localizes one centrosome to the niche-GSC interface [16]. Using the same methodology as we used to quantitate PBD-GFP localization at the niche-GSC interface, we found that Apc2 is also concentrated at the niche-GSC interface in female GSCs (4.7±3.9∶1, *n* = 20; Figure 2L) and that asymmetric Apc2 localization was disrupted in both *Rac* mutant GSC clones (1.28±0.87∶1, *n* = 12; Figure 2M) and GSCs expressing *Rac1^V12^* (1.29±0.7∶1, *n* = 23; Figure 2N). Furthermore, reduction of *Apc2* activity caused same classes of centrosome defects observed in GSCs with altered *Rac* activity (Figure 2O and 2P), as did overexpression of GFP-tagged wild-type Apc2 (Figure 2Q and 2R).

In male GSCs, the two centrosomes are asymmetric, in that the mother centrosome harbors a robust array of microtubules and remains near the niche-GSC interface, while the daughter centrosome has fewer associated microtubules and migrates around the GSC cortex to set up the plane of GSC division [20]. Staining of interphase male or female GSCs indicated that microtubule bundles are present near the niche-GSC interface (Figures S3D and 2S). Moreover, in male or female GSCs expressing Rac1^V12^, such microtubule asymmetry is abolished; microtubules are localized uniformly around the entire GSC cortex (Figures S3E and 2T). In female GSCs with reduced *Apc2* activity, a lower level of microtubules is uniformly present around the GSC cortex (Figure 2U). Therefore, we propose that Rac, acting through Apc2, controls microtubule organization to influence GSC centrosome orientation.

### GSCs with Misoriented Centrosomes Arrest at Prometaphase

Despite the necessity for *Rac* to properly localize interphase centrosomes, all mitotic spindles in *Rac-RNAi* GSCs were oriented normally, perpendicular to the niche-GSC interface with one spindle pole associated with the interface (Figure 2V). Thus, there must be a second process that functions subsequent to Rac-mediated centrosome orientation to ensure proper orientation of the GSC division plane. Cheng et al. [22] observed that if neither centrosome in male GSCs is located at the niche-GSC interface, the GSC does not undergo mitosis, but that reorientation of centrosome position allows mitotic entry, suggesting that male GSCs with misoriented centrosomes arrest in the S or G2 phase.

**Figure 3:**
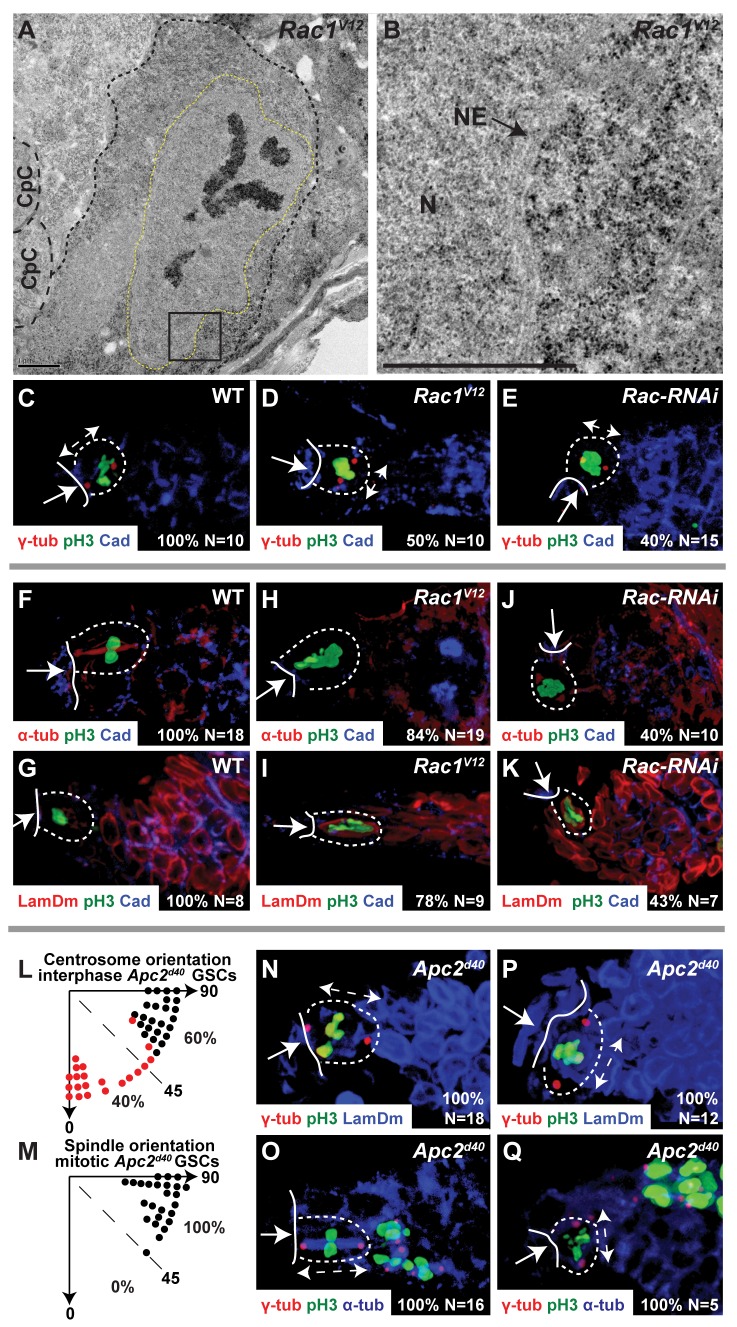
Centrosome misorientation causes GSC cell cycle arrest. (A,B) Transmission electron micrograph of a *Rac1^V12^* GSC with condensed chromosomes, intact nuclear envelope, and no mitotic spindle. (A) Small-dashed line, GSC cell membrane; large-dashed line, CpC cell membrane; dashed yellow line, GSC nuclear envelope. (B) Boxed region of (A); N, GSC nucleus; NE, GSC nuclear envelope. Scale bars, 1 µm. (C–E) Centrosome orientation in anti-pH3 stained GSCs, wild-type (C), *Rac1^V12^* (D), and *Rac-RNAi* (E). (F–K) Spindle formation (F,H,J) and nuclear envelope breakdown (G,I,K) in anti-pH3 stained GSCs, wild-type (F,G), *Rac1^V12^* (H,I), and *Rac-RNAi* (J,K). (L,M) Angle, in individual *Apc2^d40^* GSCs, between the CpC-GSC interface and the line connecting two cortical centrosomes separated by 180° (L) or mitotic spindle poles (M). Black dot, one centrosome (L) or spindle pole (M) at the CpC-GSC interface. Red dot, neither centrosome (L) or spindle pole (M) at the CpC-GSC interface. Percentage (%) of GSCs with each phenotype compared to total in the graph. (N–Q) Absolute correlation between centrosome orientation and M-phase progression in *Apc2^d40^* GSCs. (C–K, N–Q) Arrow, CpC niche; solid line, CpC-GSC interface; dashed outline, individual GSC. (C–E,N–Q) Anti-γ-tubulin, centrosomes; double headed arrow, centrosome orientation; (F,H,J,O,Q) anti-α tubulin, mitotic spindle; (G,I,K,N,P) anti-Lamin-Dm, nuclear envelope; (C–K,N–Q) anti-pH3, mitotic chromosomes. (C–K, N–Q) Percentage (%) of total (*N*) GSCs with the specific phenotype displayed in the panel.

To begin to analyze cell cycle progression in female GSCs with misoriented centrosomes, we quantitated the percentage of female GSCs that stained with an anti-phospho-Histone H3 Ser10 (pH3) antibody. The percentage of pH3-stained GSCs was higher in *Rac1^V12^*, *Rac-RNAi*, and *Apc2* GSCs than in wild-type GSCs (Table 1), suggesting that centrosome misorientation was correlated with a perturbation in the cell cycle. We then identified a *Rac1^V12^* GSC by transmission electron microscopy that had fully condensed chromosomes, but had an intact nuclear envelope and no observable mitotic spindle (Figure 3A and 3B), indicating that this GSC was arrested in a prometaphase state.

**Table 1:** Percentage of WT, *Rac*, and *Apc2* GSCs in Mitosis.

Genotype	% GSCs with anti-pH3 Staining	% pH3-Stained GSCs without Spindle	% pH3-Stained GSCs without NEBD
WT	1.1 (*n* = 554)	0 (*n* = 18)	0 (*n* = 8)
*Rac1^V12^*	5.4 (*n* = 598)	84 (*n* = 19)	78 (*n* = 9)
*Rac-RNAi*	3.3 (*n* = 522)	40 (*n* = 10)	43 (*n* = 7)
*Apc2^d40^*	2.5 (*n* = 444)	32 (*n* = 22)	42 (*n* = 12)

pH3, anti-phosphohistone H3 antibody; NEBD, nuclear envelope breakdown.

Immunofluorescence confirmed the existence of this prometaphase arrest in GSCs of all three genotypes: *Rac1^V12^*, *Rac-RNAi*, and *Apc2*. Wild-type GSCs enter mitosis with a pair of correctly oriented centrosomes (Figure 3C), while centrosomes in some *Rac* gain-of-function and loss-of-function GSCs remain misoriented even after mitotic onset (Figure 3D and 3E). All pH3-stained wild-type GSCs had a correctly oriented mitotic spindle that stained with an anti-α-tubulin antibody (Figure 3F), and had undergone nuclear envelope breakdown, as evidenced by absence of staining with an anti-Lamin-Dm antibody (Figure 3G). Conversely, approximately 80% of *Rac1^V12^* (Figure 3H and 3I), 45% of *Rac-RNAi* (Figure 3J and 3K), and 25% of *Apc2* pH3-stained GSCs (Figure S4A–D) lacked a mitotic spindle and had not undergone nuclear envelope breakdown. Interestingly, we also observed cell cycle arrest in early germline cysts of all three genotypes (Figure S4G–L, compared to wild-type Figure S4E–F), suggesting that a similar cell cycle arrest mechanism is present in cystocyte divisions. Thus, like male GSCs, female GSCs with misoriented centrosomes can undergo a cell cycle arrest; however, the arrest in female GSCs occurs at a later stage in the cell cycle, after chromosome condensation in prometaphase, compared to the S/G2 arrest in male GSCs.

We then determined whether the observed prometaphase cell cycle arrest was statistically correlated with centrosome misorientation. Although centrosome position in interphase *Apc2* GSCs was randomized (53%, *n* = 45, Figure 3L), all mitotic spindles in *Apc2* GSCs (*n* = 25, *p*<0.001, chi-square test) were oriented normally with one pole adjacent to the niche-GSC interface (Figure 3M and Figure S4A). All pH3-stained *Apc2* GSCs lacking a nuclear envelope (*n* = 18, *p*<0.001, Figure 3N) or with a mitotic spindle (*n* = 16, *p*<0.001, Figure 3O) had two centrosomes aligned perpendicular to the niche-GSC interface with one centrosome adjacent to the interface. Conversely, all pH3-stained *Apc2* GSCs with an intact nuclear envelope (*n* = 12, *p*<0.001, Figure 3P) or lacking a mitotic spindle (*n* = 5, *p*<0.05, Figure 3Q) had misoriented centrosomes neither of which was at the niche-GSC interface. The absolute correlation between the two phenotypes indicates that centrosome misorientation very likely causes the prometaphase arrest.

These data suggest that centrosomes are mobile in *Apc2* mutant GSCs, or in *Rac* mutant GSCs that lack cortical localization of Apc2, and if one centrosome becomes oriented with the niche-GSC interface, mitosis can proceed, which is similar to the function of the cell cycle arrest in male GSCs [22]. We hypothesize that uniform cytoplasmic localization of Apc2 in *Rac1^V12^* GSCs traps centrosomes, preventing centrosome movement and alignment with the niche-GSC interface, and thereby causing a higher percentage of GSC arrest (Table 1). Furthermore, none of the three mitotic spindles we observed in *Rac1^V12^* GSCs were perpendicular to the niche-GSC interface (not shown), suggesting that *Rac1^V12^* GSCs eventually overcome the prometaphase arrest and undergo mitosis with misoriented centrosomes.

To determine whether interactions between CpCs and GSCs are essential to invoke cell cycle arrest, we examined mitotic divisions in *TkvAct* ovaries, in which all germ cells have high levels of BMP signaling and adopt a GSC-like morphology. In these ovaries, all pH3-stained GSCs had a mitotic spindle. Moreover, the spindle orientation of GSCs in the CpC niche was perpendicular to the niche-GSC interface (Figure S4M and S4O, *n* = 6, quantitated in Figure S4P), while the spindle orientation of GSC-like cells outside the niche was random with respect to the niche-GSC interface (Figure S4M and S4N, *n* = 29, quantitated in Figure S4P), suggesting the interaction between the CpC niche and GSCs, and not high levels of BMP signaling, is essential to orient the GSC division plane. In conclusion, we have shown that a Rac/Apc2-independent cell cycle arrest functions together with the Rac/Apc2-dependent localization of interphase centrosomes to ensure the invariant division plane of female GSCs.

### Active Rac Elevates BMP Signaling in GSCs

The two primary characteristics of the asymmetric self-renewal division of the GSC are the essentially invariant orientation of the plane of GSC division and a sharp decrease in BMP signaling between the GSC and Cb. Above, we demonstrated that asymmetrically activated Rac is necessary for GSC centrosome localization. However, because Rac is active only at the niche-GSC interface, it is possible that upon GSC division activated Rac is segregated only to the daughter that remains a GSC. Therefore, we assayed whether Rac could also elevate BMP signaling specifically in GSCs.

**Figure 4:**
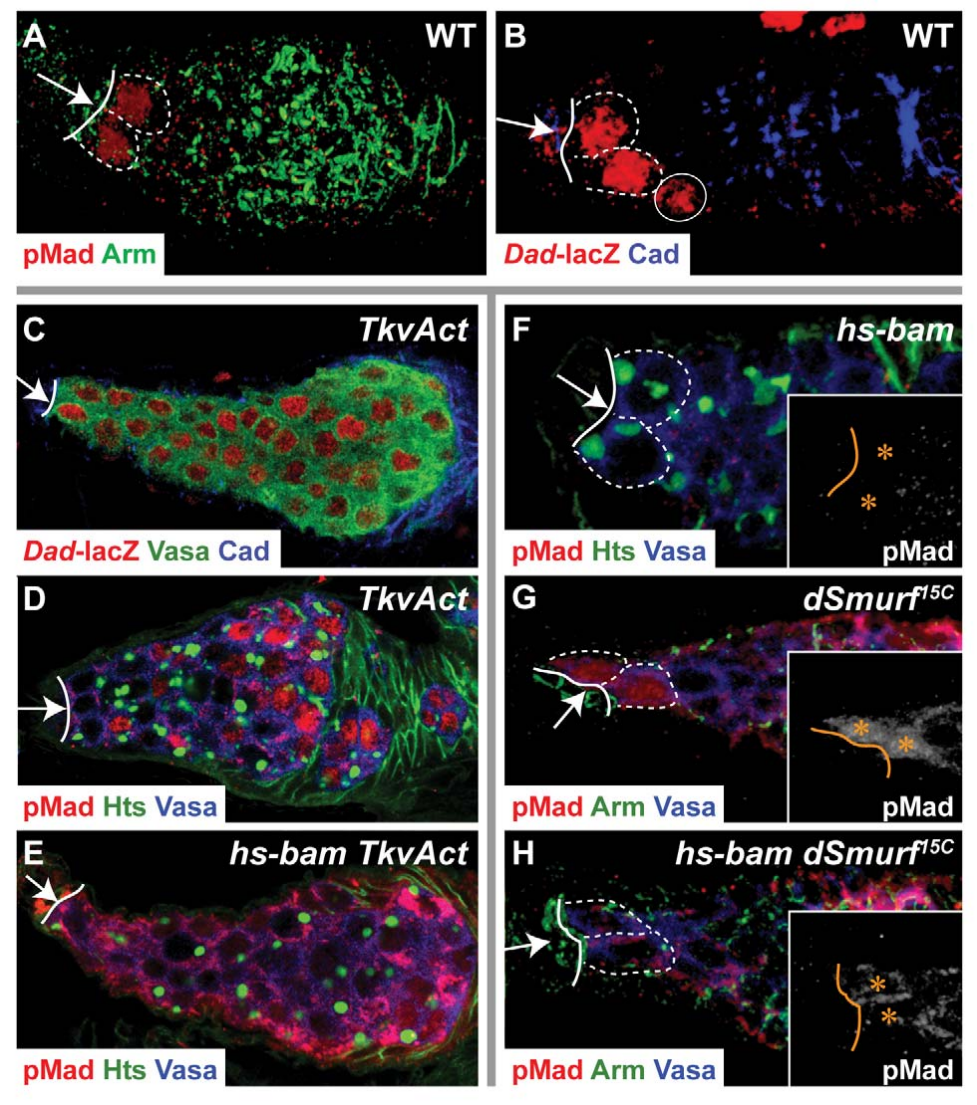
Bam blocks nuclear accumulation of pMad and dSmurf degrades cytoplasmic pMad. (A,B) BMP signaling in wild-type germaria visualized with anti-pMad antibody (A) or by *Dad*-*lacZ* expression (B). (C) A tumorous *TkvAct* germarium with BMP signaling in all germ cells, as evidenced by *Dad*-*lacZ* expression. (D–H) Flies of genotype *TkvAct* (D), *hs-bam TkvAct* (E), *hs-bam* (F), *dSmurf^15C^* (G), or *hs-bam dSmurf^15C^* (H) were subject to a 2-h heat shock at 37° and a 4-h recovery at room temperature. (D) A *TkvAct* ovariole with pMad staining in germ cell nuclei, indicated by absence of Vasa staining. (E) A *hs-bam*; *TkvAct* ovariole with decreased nuclear localization of pMad and increased cytoplasmic pMad. (F) A *hs-bam* germarium with no pMad staining visible in GSCs. (G) A *dSmurf^15C^* germarium with both nuclear and cytoplasmic pMad staining in GSCs, and cytoplasmic pMad staining in Cbs and germline cysts. (H) A *hs-bam*; *dSmurf^15C^* germarium with loss of pMad nuclear staining but retention of cytoplasmic pMad staining in GSCs. (A–H) Arrow, CpC niche; solid line, CpC-GSC interface. (A–B,F–H) Dashed outline, individual GSC. (B) Solid circle, a Cb with low levels of β-galactosidase staining. (F–H) Inset: white, anti-pMad staining; asterisk, GSC nucleus.

The sharp decrease in BMP signaling from GSCs to Cbs (Figure 1A) can be visualized by three methodologies [9]–[11]. First, an antibody against the active phosphorylated form of the BMP signal transducer Mad (pMad) specifically stains the GSCs (Figure 4A). Second, there are high levels of *lacZ* expression from an enhancer trap in the BMP target gene *Dad* (*Dad-lacZ*) in GSCs (Figure 4B). Given the specificity of the pMad staining (Figure 4A), the lower level of anti-β-galactosidase staining in the Cb (Figure 4B) likely results from perdurance of the β-galactosidase protein. Third, GFP expression from a *bam* transcriptional reporter (*bam*-GFP) is not present in GSCs, but is present at extremely low levels in Cbs and high levels in dividing cyst cells (Figure S5C). While the great majority of ovarioles expressing *Rac1^V12^* displayed a normal pattern of BMP signaling as measured by *Dad-lacZ* expression, BMP signaling in 1% of ovarioles (*n* = 200) was expanded to encompass the whole germarium (not shown), suggesting that activated Rac could promote germline BMP signaling, but that other factors largely blocked its activity.

We thus reasoned that expression of activated Rac in the background of a loss of a negative regulator of BMP signaling in the germ line could increase the penetrance of the BMP signaling defect. Previously, we identified two genes, *bam* and the E3 ubiquitin ligase *dSmurf*, that act redundantly to downregulate germline BMP signaling. While Bam antagonizes BMP signaling downstream of the BMP receptor Thickveins [10], dSmurf targets multiple components of the BMP signaling pathway for degradation: in the wing discs, dSmurf ubiquitinates pMad [26]; and in Cbs and posterior germ cells, dSmurf interacts with the Fused (Fu) kinase to ubiquitinate the Tkv receptor [27].

To further characterize the action of Bam, we first assayed its function in the germaria of *TkvAct* females. All germ cells in *TkvAct* germaria expressed *Dad-lacZ* (Figure 4C). In control heat-shocked *TkvAct* germaria, nuclear pMad was present in 48% of individual germ cells 4 h after heat shock (*n* = 732 cells in six germaria) (Figure 4D). Conversely, in *TkvAct* germaria with heat shock-induced Bam expression, nuclear pMad was present in only 5% of germ cells 4 h after heat shock (*n* = 668 cells in five germaria). Strikingly, in these germaria pMad was predominantly localized throughout the germline cytoplasm (Figure 4E). In contrast, heat-shock-induced expression of Bam in wild-type GSCs resulted in rapid loss of pMad from the GSC (Figure 4F). We hypothesized that in wild-type GSCs, unlike the GSC-like cells in *TkvAct* germaria, cytoplasmic pMad is rapidly degraded, possibly by the action of DSmurf. While pMad was present only in the nucleus of wild-type GSCs subject to a control heat shock (not shown), in *dSmurf* females subject to the same control heat shock, pMad was observed in both the nucleus and cytoplasm of the GSC (Figure 4G), demonstrating that DSmurf is active in GSCs. Heat-shock-induced expression of *bam* in *dSmurf* females resulted in the accumulation of pMad in the cytoplasm of GSCs (Figure 4H). Thus, we conclude Bam blocks BMP signaling by preventing the nuclear accumulation of pMad.

**Figure 5:**
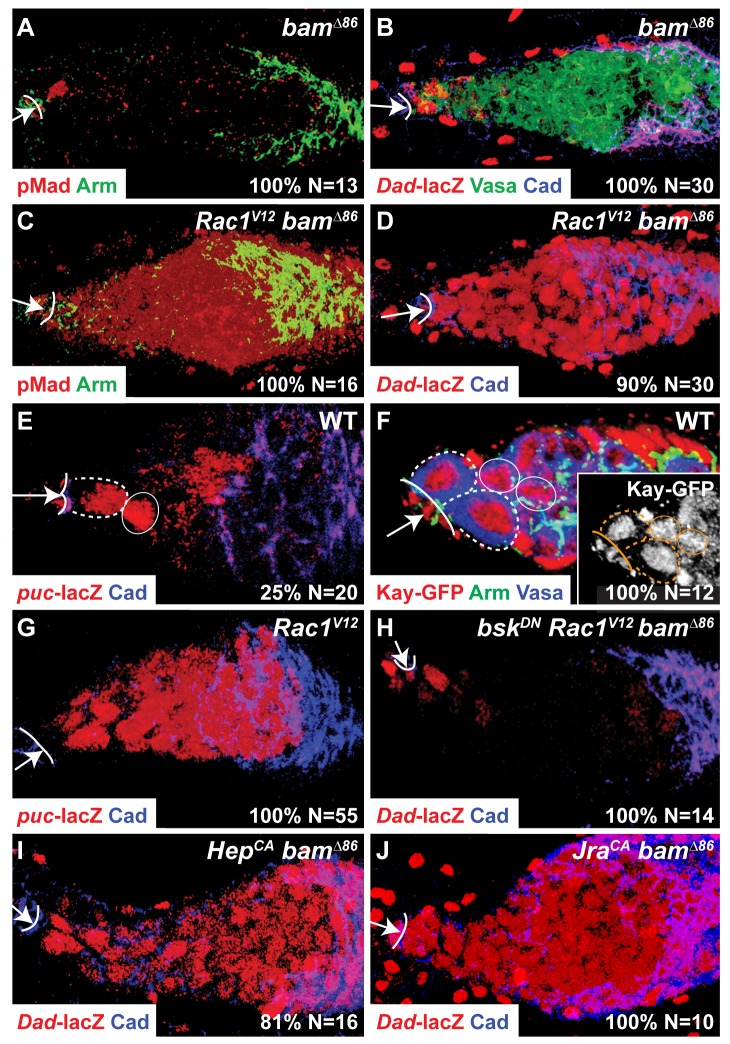
Rac promotes germline BMP signaling by activating the JNK pathway. (A–D) BMP signaling in germaria of *bam^Δ86^* females (A,B), and *bam^Δ86^* females with germline expression of *Rac1^V12^* (C,D). (E,G) JNK activity, visualized by *puc*-*lacZ* expression, in one GSC of a wild-type germarium and in its descendent Cb, probably as a result of perdurance of β-galactosidase (E), and in all germ cells in a germarium of a female with germline expression of *Rac1^V12^* (G). (F) Kayak-GFP expression in anterior germarium of wild-type female. Inset: white, anti-GFP staining. (H–J) BMP signaling in germaria of a *bam^Δ86^* female with (H) germline expression of *Rac1^V12^* and dominant negative form of Bsk (*bsk^DN^*), (I) germline expression of constitutively active form of Hep (*hep^CA^*), or (J) germline expression of a constitutively active form of Jra (*Jra^CA^*). (A–J) Arrow, CpC niche; solid line, CpC-GSC interface. (E,F) Dashed outline, individual GSC; solid circle, individual Cb. (A–J) Percentage (%) of total (*N*) germaria with the specific phenotype displayed in the panel.

Based on the above data, we reasoned that a null allele of *bam*, *bam^Δ86^*, could be a sensitized background in which to assay the effect of Rac activity on germline BMP signaling. While *bam* mutant ovarioles have a normal pattern of BMP signaling (Figure 5A and 5B) due to the action of *dSmurf* [10], expression of *Rac1^V12^* in the *bam* mutant background caused expansion of BMP signaling throughout the germarium, shown by either anti-pMad antibody staining (Figure 5C) or *Dad-lacZ* expression (Figure 5D). Expression of *Rac1^V12^* also caused a spatial expansion of BMP signaling in *dSmurf* mutant ovarioles, although to a lesser extent (not shown). Thus, activated Rac promotes BMP signaling in the germarium.

Because Rac can elevate the activity of the JNK pathway in multiple developmental contexts (e.g., embryonic dorsal closure in *Drosophila* [28]), we examined whether Rac acts through the JNK pathway to promote BMP signaling in GSCs. The JNK pathway comprises a kinase cascade, JNKKK→JNKK→JNK, that leads to phosphorylation of the transcription factor c-Jun [29], heterodimer formation of pJun and Fos, and transcriptional activation of multiple genes, including the phosphatase *puckered* (*puc*) [30], which acts as a negative regulator of the JNK pathway by dephosphorylating the JNK homolog Basket (Bsk). To determine whether Rac activates the JNK pathway in GSCs, we assayed expression of a *puc-lacZ* enhancer trap. In 25% (*n* = 20) of ovarioles, *puc-lacZ* was specifically expressed in a GSC and, likely due to perdurance of β-galactosidase protein, in a Cb (Figure 5E). Thus, the JNK pathway is active in GSCs, but other factors may modulate its activity. Because the *Drosophila* homolog of Fos, Kayak, identified by a functional GFP protein trap, always localized to the nucleus of GSCs, Cbs, and early cyst cells (Figure 5F), Rac-mediated activation of the JNK pathway in GSCs likely depends on nuclear import of active phosphorylated c-Jun.

Unlike wild-type ovarioles, in ovarioles expressing *Rac1^V12^*, *puc-lacZ* was always expressed throughout the germarium (Figure 5G), indicating that Rac can activate the JNK pathway in the germ line. Coexpression of a dominant negative form of *bsk*, *bsk^DN^*, with *Rac1^V12^* in the *bam* background completely suppressed ectopic BMP signaling (Figure 5H, compare to Figure 5D), indicating that Rac-mediated expansion of BMP signaling is completely dependent on JNK activity. Moreover, expression of constitutively active forms of the *Drosophila* homologs of JNKK, *hemipterous* (*Hep^CA^*) [31], and the transcription factor c-Jun, *Jra* [32], in the *bam* background also expanded BMP signaling (Figure 5I and 5J). Taken together, these data suggest that the Rac-activated JNK pathway controls one or more transcriptional targets to promote BMP signaling in the GSC.

We next wished to determine whether Rac/JNK activity is necessary for BMP signaling in GSCs. However, there was no obvious difference in pMad staining or *bam*-GFP expression in *Rac* mutant (Figure S5B and S5D) or *bsk* mutant GSCs (Figure S5E) compared to control GSCs (Figures S5A and S5C), suggesting that other genes act in parallel with the Rac/JNK pathway to promote BMP signaling in GSCs.

**Figure 6:**
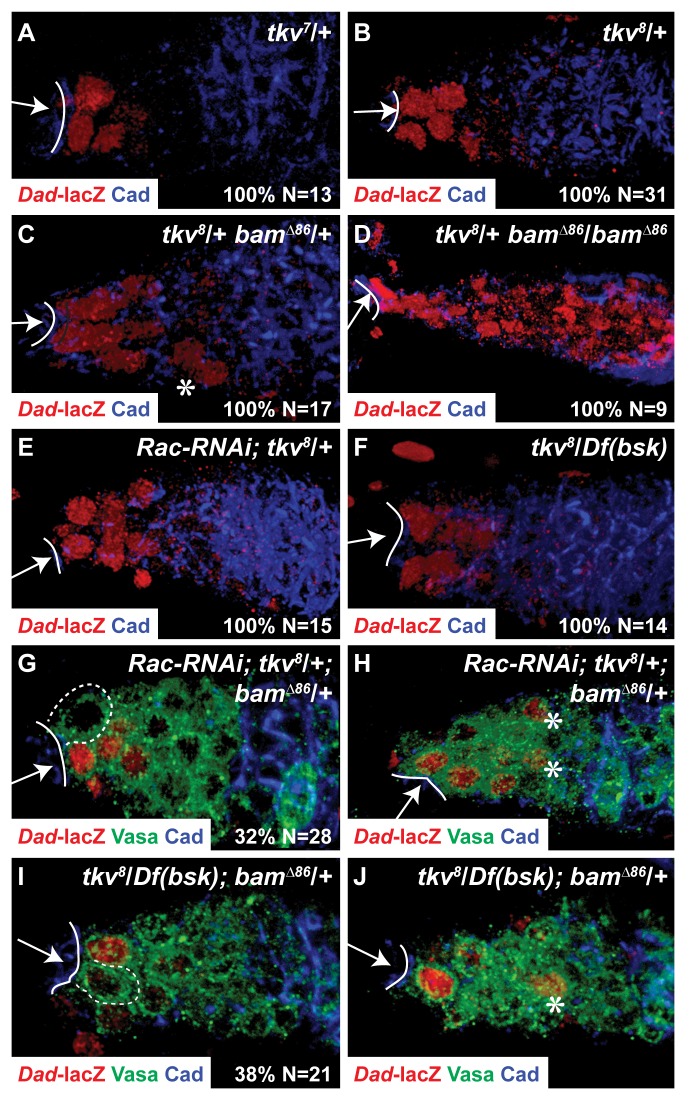
A sensitized genetic background reveals Rac/JNK function in promoting BMP signaling in GSCs. (A–D) The Dpp receptor Tkv constrains ligand diffusion. Although heterozygosity for the kinase-dead allele *tkv^7^* (A) causes no difference in *Dad*-*lacZ* expression compared to the wild-type germarium (Figure 4B), heterozygosity for the protein null allele *tkv^8^* (B) increases the number of germ cells expressing *Dad*-*lacZ*. (C) Heterozygosity for *bam* further expands BMP signaling in *tkv^8^*/+ germaria, as evidenced by *Dad-lacZ* expressing germ cells more posterior in the germarium (asterisk). All niche-associated GSCs had *Dad*-*lacZ* expression. (D) Complete loss of *bam* causes *Dad*-*lacZ* expression to expand throughout the germaria of *tkv^8^*/+ females. (E–F) Germline expression of *Rac-RNAi* (E) or heterozygosity for *bsk* (F) slightly expands *Dad*-*lacZ* expression in *tkv^8^*/+ germaria, but all niche-residing GSCs have *Dad*-*lacZ* expression. (G–J) A sensitized background reveals the role of Rac and JNK activity in promoting BMP signaling in GSCs. Germaria from *tkv^8^*/+; *bam*/+ females with germline expression of *Rac-RNAi* (G–H) or heterozygous for *bsk* (I–J) in which a niche-associated GSC (dashed outline in G,I) does not have *Dad*-*lacZ* expression, but *Dad*-*lacZ* expressing germ cells are present more posterior in the germarium (asterisks in H,J). (G,H) are different confocal sections from a single germarium of the specified genotype, as are (I,J). (A–J) Arrow, CpC niche; solid line, CpC-GSC interface. (A–F,G,I) Percentage (%) of total (*N*) germaria (A–F) or GSCs (G,I) with the specific phenotype displayed in the panel.

We then sought to identify a genetic background in which the loss of Rac/JNK signaling could have phenotypic consequences on BMP signaling in the GSC. We reasoned that if a greater number of germ cells were exposed to BMP ligands secreted by the CpCs, then the amount of ligand per GSC could become limiting. In such a background, the ability of Rac/JNK to promote BMP signaling in the GSCs might become necessary. Because the Tkv receptor on the cell surface can block ligand diffusion by acting as a sink for BMP ligands [33], we examined the spatial extent of BMP signaling after reduction in Tkv levels. While heterozygosity for *tkv^7^*, an allele that produces a non-functional kinase with normal BMP ligand-binding ability, did not affect the number of *Dad-lacZ* positive cells (Figure 6A), heterozygosity for the protein null allele *tkv^8^* increased the number of *Dad-lacZ* positive cells (Figure 6B, compare to Figure 4B), indicating a reduction in Tkv receptor concentration, not a reduction in receptor signaling, causes an increase in the spatial extent of BMP signaling. The expansion of BMP signaling caused by *tkv^8^* heterozygosity was further enhanced in a *bam*/+ background (Figure 6C) and BMP signaling was present throughout the germarium in a *tkv^8^*/+; *bam*/*bam* background (Figure 6D, compare to Figure 5B). Thus, high levels of Tkv receptor on the GSC are critical to restrict diffusion of the Dpp ligand to ensure absence of BMP signaling in Cbs. In the wing disc, Hedgehog and BMP-mediated downregulation of the Tkv receptor near the source of Dpp ligand is necessary to permit Dpp diffusion to establish a long-range gradient of Dpp across the disc [33]–[35], indicating that modulation of receptor levels can be used either to allow or to restrict diffusion of ligands across a field of cells.

Because *tkv^8^*/+; *bam*/+ females have a larger number of germ cells with BMP signaling, it is likely that each germ cell is exposed to less Dpp ligand. Thus, this genotype could act as a sensitized background to reveal the function of Rac in promoting BMP signaling in the GSC. While BMP signaling was present in all niche-associated GSCs in *tkv^8^*/*+*; *bam*/*+* germaria (Figure 6C), in *Rac-RNAi*; *tkv^8^*/+ germaria (Figure 6E) and in *tkv^8^+/+Df(bsk)* germaria (Figure 6F), approximately one-third of *Rac-RNAi*; *tkv^8^*/*+*; *bam*/*+* GSCs (*n* = 28, *p*<0.01, chi-square test, Figure 6G) and *tkv^8^*+/+*Df(bsk)*; *bam*/*+* GSCs (*n* = 21, *p*<0.01, Figure 6I) lacked *Dad-lacZ* expression. Moreover, in the same germaria, *Dad-lacZ* staining could be present in more posterior germ cells (asterisks in Figure 6H and 6J). Thus, in a background of putative decreased ligand availability, the activity of the Rac/JNK pathway becomes necessary to ensure that all GSCs have BMP signaling.

## Discussion

**Figure 7:**
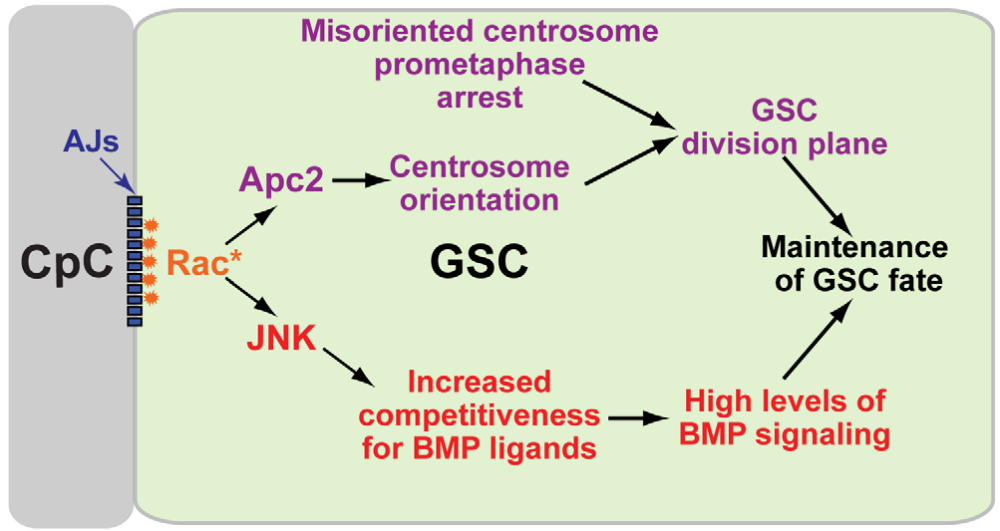
Model of Rac function in the GSC. Activated Rac (Rac*) at the CpC-GSC interface sets up an intracellular polarity within the GSC that couples the control of the plane of cell division to the response to the extracellular BMP maintenance signal. Activated Rac concentrates Apc2 at the CpC-GSC interface to orient a centrosome during interphase. The GSC will arrest in prometaphase if one centrosome is not present at the CpC-GSC interface. These two mechanisms act together to ensure that the GSC mitotic spindle is perpendicular to the niche-GSC interface. Rac also activates the JNK pathway to promote BMP signaling in the GSC, possibly by increasing competitiveness of the GSC for BMP ligands.

In this article, we have shown that the small GTPase Rac is activated at the niche-GSC interface to influence two major aspects of the asymmetric self-renewal division of the GSC (summarized in Figure 7). First, Rac activity orients the GSC division plane by localizing one interphase GSC centrosome at the niche-GSC interface. Second, Rac activity promotes BMP signaling in the GSC. Other processes act in parallel with both functions of Rac, likely to ensure the robustness of this asymmetric division. In particular, the GSC cell cycle is arrested at prometaphase if centrosomes are not localized at the niche-GSC interface. Therefore the asymmetric activation of Rac within the GSC links a mechanism that influences the plane of GSC division with a mechanism that promotes distinct fates in GSC daughters.

### Rac Is Necessary for Maximal GSC Half-Life

Certain genetic backgrounds, such as loss of BMP signaling within a GSC or loss of adherens junction in one GSC, decrease GSC half-life from greater than 4 wk to less than 1 wk [5],[6]. Conversely, our data demonstrate that Rac mutant GSCs have a half-life of about 2 wk. These data raise the question of which of the two functions of Rac has the greater effect on GSC maintenance. While *Rac* mutant GSCs lack normal centrosome orientation and can arrest in prometaphase, all *Rac* mutant GSCs divide with a normal orientation. Furthermore, in males, in which the niche is larger and can accommodate an increased number of GSCs, perturbations in *Rac* activity lead to an increase in GSC numbers, even though the GSCs have misoriented centrosomes. Taken together, these data suggest that centrosome misorientation may not markedly affect GSC half-life. Conversely, we have shown that *Rac* activity can be necessary for BMP signaling in a sensitized background in which GSCs are likely exposed to less BMP ligand. We propose that lack of Rac/JNK activity in a GSC could lessen the robustness of the GSC response to decreasing levels of BMP ligands as the fly ages [36],[37], thereby resulting in a reduction in the half-life of *Rac* mutant GSCs.

### Rac, Acting Together with a Prometaphase Cell Cycle Arrest, Orients the Plane of GSC Division

Our data establish that centrosome behavior is controlled similarly in both sexes. Specifically, we used both fixed and live imaging to demonstrate that, similar to male GSCs [16], one centrosome in female GSCs is positioned at the niche-GSC interface throughout the cell cycle, and that the second centrosome migrates around the GSC cortex to ultimately orient the plane of GSC division. Moreover, similar to male GSCs [16], in female GSCs, Apc2 is concentrated at the niche-GSC interface, and Apc2 concentration promotes an asymmetric distribution of cytoplasmic microtubules at the niche-GSC interface. Lastly, we demonstrated that Rac activity in both sexes is necessary for proper centrosome orientation and microtubule asymmetry. Because ectopic Rac activity can be sufficient to direct Apc2 throughout the GSC, the concentration of Apc2 at the niche-GSC interface is not likely to be mediated solely by direct physical interactions between Apc2 and components of the adherens junctions.

While Rac acts through Apc2 to orient interphase centrosomes, female GSCs with mispositioned centrosomes arrest at prometaphase with lack of nuclear envelope breakdown and mitotic spindle formation. Cheng et al. also reported a cell cycle arrest invoked by centrosome misorientation in male GSCs [22]. Their study showed that, while male GSCs with misoriented centrosomes rarely divided, reorientation of GSC centrosomes led to rapid mitotic entry. However, the GSC cell cycle appears to arrest at an earlier stage in males, the S or G2 phases, than it does in females, prometaphase, and it will be interesting to compare the mechanisms responsible for cell cycle arrest in each sex.

### Rac Is One of Multiple Mechanisms That Ensure Differential BMP Signaling between GSCs and Cbs

To ensure maximal fertility, the mechanisms responsible for differential BMP signaling between the GSC and its sister Cb must be extremely robust. BMPs are secreted from the CpCs of the niche, and BMP signaling is only observed in GSCs. Since, in other developmental contexts, BMP ligands are able to diffuse over a field of cells, additional mechanisms must modulate BMP ligand distribution, reception, and/or signal transduction to ensure the binary pattern of BMP signaling between the GSC and the Cb.

Our data indicate that local activation of Rac promotes BMP signaling in the GSC. Rac activates the JNK pathway, and we propose that activation of the JNK pathway increases the ability of the GSCs, compared to their sister Cb cells, to compete for BMP ligands secreted by the CpCs. While Rac/JNK activity is not absolutely required for BMP signaling in the GSC, we identified a sensitized genotype (*tkv*/+; *bam*/+) where both Rac and JNK activity are necessary to ensure the GSCs are always positive for BMP signaling, thereby demonstrating that both genes are active in the GSC to promote BMP signaling. Perhaps surprisingly, activation of Rac or the JNK pathway throughout the germarium of *bam* mutants was sufficient to cause all germ cells to have BMP signaling. It is possible that substantial BMP ligand is present in the posterior region of the germarium, but wild-type germ cells are not competent to respond to endogenous ligand. In this scenario, the lack of *bam* coupled with ectopic JNK activity allows the germ cells to receive and respond to endogenous ligand.

Additional mechanisms also contribute to the differential response of the GSCs and Cbs to BMP ligands. First, Tkv receptors likely act as a sink to sequester the Dpp ligands and block their ability to diffuse within the germarium. We showed that reduction of *tkv* dose permits additional germ cells near the niche to receive BMP signaling, an effect that is increased in genetic backgrounds in which *bam* is reduced. Second, the heparin sulfate proteoglycan Dally is expressed in the CpCs and both restricts ligand diffusion and acts as a potential trans co-receptor for BMPs [38],[39]. GSCs send out an EGF signal that blocks Dally expression in the escort cells, thereby concentrating BMPs at the CpC surface [40]. Thus, at least three mechanisms have been identified that either modulate BMP ligand distribution or the responsiveness to BMP ligands, near or within the niche to promote differential BMP signaling between GSCs and Cbs.

Conversely, a similar number of mechanisms have been identified that restrict the ability of the Cbs and their descendants to receive or respond to a BMP signal. We have shown the BMP target gene *bam* and the ubiquitin ligase *dSmurf* function redundantly in Cbs and early cysts to downregulate BMP signaling downstream of receptor activation [10]. Bam downregulates BMP signaling by blocking nuclear localization of pMad, while Smurf degrades Mad [26] and acts together with Fused to degrade activated Tkv receptors [27]. Moreover, miR-184 down-regulates the BMP receptor Saxophone in posterior cyst cells [41]. Lastly, Brat mediated translational repression of target genes, including Mad and cMyc, also promotes Cb differentiation [42]. Thus, mechanisms that promote BMP signaling in the GSCs act in concert with mechanisms that repress BMP signaling in Cbs and early cysts. These data highlight the complexity and genetic redundancy necessary to ensure a robust pattern of asymmetric BMP signaling between the GSC and Cb.

### Adherens Junctions Likely Play Multiple Roles in the Control of GSC Behavior

In both sexes adherens junctions have been proposed to anchor GSCs to their cellular niche. However, there is an increasing appreciation that adherens junctions play additional roles in GSC maintenance. Previous work indicated that adherens junctions localize Apc2 to orient the male GSCs centrosome [16],[21]. Our data indicate that Rac mediates all aspects of adherens junction function in orienting the GSC centrosome including Apc2 localization, suggesting that adherens junctions could also lead, directly or indirectly, to Rac activation within the GSC. Other data indicate that differences in the levels of adherens junctions can mediate GSC competition for niche occupancy [43], and that, in male GSCs, the presence of adherens junctions leads to asymmetric reception of the BMP signal within the GSC [44]. Thus, adherens junctions play far more than a structural role in the asymmetric self-renewing divisions of the GSC.

## Materials and Methods

### *Drosophila* Strains and Culture

Fly stocks were maintained at 18–25°C on standard cornmeal molasses medium. Some fly stocks were obtained from the *Drosophila* Stock Center, the National Institute of Genetics, or the Yale GFP Protein Trap Database.

### Immunohistochemistry

Ovaries were dissected in PBS, fixed for 15 min in 4% paraformaldehyde solution (Electron Microcopy Services) with PBTF (1.5 mM NaH_2_PO_4_, 3.5 mM Na_2_HPO_4_, 65 mM NaCl and 0.062% Tween-20), washed six times with PBT, blocked for 1 h in 500 µl NBT (5% Normal Goat Serum in PBT), and incubated in the appropriate dilution of the primary antibody in NBT overnight at 4°C or at room temperature for 2–4 h. After incubation, ovaries were washed with PBT, blocked for 1 h in NBT, and incubated with secondary antibody. Ovaries were washed with PBT and were mounted in Prolong Gold antifade reagent (Invitrogen) prior to imaging.

### Localization of Activated Rac in the GSC

Adult females of genotype *P{Gal4-Hsp70}*/+; *P{UASp-PakPBD::eGFP}*/+ were grown at 25°C for 2 d and transferred into pre-warmed vials at 37°C for 1 h. Three hours after heat shock, ovaries were dissected and stained with anti-GFP, anti-Vasa, and anti-Armadillo antibodies (for non-clonal analysis) or anti-GFP, anti-β-gal, and anti-DE-Cad antibodies (for clonal analysis). The expression of PakPBD::eGFP varied between individual ovarioles. In addition, GSCs were less sensitive to heat shock than more posterior germ cells. Only germaria in which anti-GFP staining in the GSCs was visible and was in a punctate, as opposed to uniform, pattern were assayed by ImageJ.

### Electron Microscopy

Ovaries from flies of genotype *P{UASp-Rac1^V12^}*/+*; P{Gal4::VP16-nos.UTR}*/+ were removed into 2% glutaraldehyde in 0.1 M Na Cacodylate buffered at pH 7.4 at 25°C for 10 min and were placed on ice for 40 min. They were rinsed in cold buffer and then were post-fixed in 2% OsO_4_ in 0.1 M Na Cacodylate for 2 h at 4°C. After a cold-water wash, they were stained in 1% uranyl acetate overnight at 4°C. Ovaries were dehydrated in ethanol and then embedded in epoxy resin (DER 732/332 mixture). Serial thin sections were stained with uranyl acetate and lead citrate and viewed in a Tecnai F30 EM at 300 kV and photographs taken with a Gatan digital camera.

## Supporting Information

Figure S1 *Rac1* is expressed in the germarium and activated at the CpC-GSC interface. (A) A *Tkv-Act* ovariole hybridized with a *Rac1* antisense probe showing *Rac1* transcription in three GSC-like cells. (B) A germarium with *Rac* mutant (*Rac1^J10^ Rac2^Δ^ Mtl^Δ^*/*Rac1^J10^ Rac2^Δ^+*) germline clones, marked by absence of *lacZ* expression, produced by mitotic recombination. Dashed outline, *Rac* mutant GSC; solid outline, *Rac* mutant Cb and cyst cells. (C–E) Anti-human Rac1 antibody specifically recognizes *Drosophila* Rac proteins. Identical staining and image processing of a wild-type ovariole (C), an ovariole from female with germline expression of Rac1^V12^ (D), and an ovariole from female with germline expression of RNAi against *Rac1* and *Rac2* (E). (F–H) After mild heat shock, PBD-GFP is localized to the CpC-GSC interface in GSCs. Posterior germ cells were more sensitive to heat shock and usually expressed PBD-GFP uniformly throughout the cytoplasm. (I) Quantitation of the asymmetry of PBD-GFP localization as a ratio of anti-GFP staining at the cortex of the CpC-GSC interface (area enclosed by dashed orange line) to the remainder of the GSC cytoplasm (area enclosed by solid white line) after subtraction of background levels in GSC nucleus (area enclosed by dotted white line). (J–L) PBD-GFP specifically recognizes active Rac in the GSCs. (J) After mild heat shock, GFP alone is localized uniformly throughout the cytoplasm. (K) Mild heat shock was used to drive expression of Rac1^V12^ and PBD-GFP in the germ line. PBD-GFP is localized to puncta throughout the *Rac1^V12^* GSCs. (L) PBD-GFP is localized to the CpC-GSC interface in females with reduced *Cdc42* activity (*Cdc42^4/6^*). (A–H,J–L) Arrow, CpC niche. (B–H,J–L) Solid line, CpC-GSC interface. (F–H,J–L) Dashed outline, individual GSC. Inset; white, anti-GFP staining. (F–H,L) Arrowhead, asymmetrically localized PBD-GFP in GSCs.
(TIF)

Figure S2 Time series of GSC centrosome migration in a living germarium. (A) Centrosomes, marked by arrowheads, are visualized by a GFP-tagged pericentrosomal protein Centrosomin (GFP-Cnn, green) under the control of germline-specific Nos-Gal4 driver. The nuclei of the somatic niche cells (and to a lesser extent, the undifferentiated anterior germline cells) are marked by a living-cell DNA dye (Draq5-Cy5, red). The boundary of Cnn-GFP and bright Draq5 staining delineates the CpC-GSC interface. The first image displays both channels and demarcates each GSC with dashes; each subsequent image displays only the Cnn-GFP channel. The niche-GSC interface is marked by a solid line and in the first image by an arrow. The series encompasses 28 min; the time of each image is listed. The germarium has two GSCs in focus. The centrosomes in the top GSC (open arrowheads) are positioned perpendicular to the niche GSC interface and do not change position. The centrosomes in the bottom GSC are marked with closed arrowheads: one centrosome is at the niche-GSC interface (closed orange arrowhead); the other is completing its migration around the cortex (closed green arrowhead). In the last image, a closed red arrowhead documents the starting position of the migrating centrosome.
(TIF)

Figure S3 *Rac* activity controls centrosome position in male GSCs. (A) A male testes with GSCs around their cellular niche, the hub (asterisk). One centrosome in the great majority of wild-type male GSCs is present at the niche-GSC interface. (B–C) Defects in centrosome position in male *Rac* RNAi (B) or in *Rac1^V12^* (C) GSCs. Two male *Rac* RNAi GSCs (B) or two male *Rac^V12^* GSCs (C) in which neither centrosome is at the niche-GSC interface. (D–D′) In wild-type interphase male GSCs, microtubules are organized as a bundled network near the hub-GSC interface. (E–E′) In *Rac1^V12^*-expressing male GSCs, a microtubule network is present uniformly around the GSC cell cortex. (A–E) Anti-Vasa, germline cytoplasm; anti-DE Cadherin, adherens junctions among hub cells and between hub cells and GSCs. (A–C) Anti-γ-tubulin, centrosomes. (D,D′,E,E′) Anti-α-tubulin, cytoplasmic microtubules. (A–E) Asterisk and dashed line, hub cells; dotted outline, individual GSC. (A–C) Percentage (%) of scored (*N*) GSCs with the phenotype represented in the panel; arrowhead, centrosome.
(TIF)

Figure S4 Mitotic phenotypes of *Apc2* and *TkvAct* GSCs and *Rac1^V12^*, *Rac-RNAi* and *Apc2* early germline cysts. (A–D) Anti-pH3 stained mitotic *Apc2* mutant GSCs. Most pH3 stained *Apc2* GSCs have a mitotic spindle with one spindle pole adjacent to the CpC-GSC interface (A) and have undergone nuclear envelope breakdown (C). Some pH3 stained *Apc2* GSCs lack a mitotic spindle (B) and have not undergone nuclear envelope breakdown (D). (E–F) Mitotic divisions of wild-type four-cell cysts are synchronous and are marked by condensed chromosomes with mitotic spindle formation (E) and nuclear envelope breakdown (F). (G–L) Mitotic four-cell cysts of *Rac1^V12^* (G,H), *Rac-RNAi* (I,J), or *Apc2* (K,L) with anti-pH3 staining that do not have mitotic spindles (G,I,K) and have not undergone nuclear envelope breakdown (H,J,L). (M–O) Mitotic GSCs and GSC-like cells in *TkvAct* germaria. While GSCs adjacent to the CpCs niche divide with an invariant division plane (M,O), the division plane of GSC-like cells not at the niche is randomized with respect to the CpC-GSC interface (M,N). (P) Quantification of spindle orientation of dividing niche-residing GSCs and non-niche-associated GSC-like cells in *TkvAct* germaria. Dot, angle between the CpC-GSC interface and spindle orientation. Cyan dot, dividing GSCs at the CpC niche. Magenta dot, dividing GSC-like cells outside the CpC niche. *N*, number scored of each of class of GSC. (A–O) Arrow, CpC niche. (A–D,M–O) Solid line, CpC-GSC interface. (A–D) Dashed outline, individual GSC. (E–L) Dashed enclosure, a dividing four-cell cyst. (M–O) Dashed outline, mitotic GSC at the CpC niche; dotted outline, mitotic GSC-like cell outside the niche. (A,M–O) Double-headed arrow, orientation of mitotic spindle. (A–D) Percentage (%) of total (*N*) GSCs with the specific phenotype displayed in the panel.
(TIF)

Figure S5 Loss of *Rac* or JNK activity does not significantly reduce BMP signaling in GSCs. (A) Equivalent levels of anti-pMad staining between GSCs (dashed circles) in a control *Rac^J10^ Rac2^Δ^ Mtl^Δ^*/+++ germarium. There was 20.0%±13.6% (*n* = 8) variability in pMad staining between pairs of GSCs in control germaria. (B) Equivalent levels of anti-pMad staining between one *Rac^J10^ Rac2^Δ^ Mtl^Δ^*/+++ GSC (dashed circle) and one *Rac^J10^ Rac2^Δ^ Mtl^Δ^*/*Rac^J10^ Rac2^Δ^*+GSC (dotted circle, marked by lack of *lacZ* expression) after mitotic recombination in a heterozygous female. Homozygous *Rac* GSCs had on average a 12.8% greater intensity of pMad staining than heterozygous *Rac* GSCs (*n* = 17), within the normal range of variability. (C) Lack of *bam*-GFP expression in a GSC (dashed circle) and extremely low levels of expression in Cbs (solid circles) in a control *Rac^J10^ Rac2^Δ^ Mtl^Δ^*/+++ germarium. *bam*-GFP expression is high in early cysts posterior to Cbs. (D) Lack of *bam-GFP* expression in a *Rac^J10^ Rac2^Δ^ Mtl^Δ^*/*Rac^J10^ Rac2^Δ^*+GSC (dashed circle, marked by lack of *lacZ* expression) and extremely low expression in a *Rac* Cb (solid circle, marked by lack of *lacZ* expression) after mitotic recombination in a heterozygous female. (E) Loss of *bsk* does not reduce BMP signaling in GSCs. Equivalent levels of anti-pMad staining between a *bsk^flp147E^*/+ GSC (dashed circle) and a *bsk^flp147E^* GSC (dotted circle) after mitotic recombination. (A–E) Arrow, CpC niche; solid line, CpC-GSC interface. (A–B,E) Inset: white, anti-pMad staining. (C–D) Inset: white, anti-GFP staining.
(TIF)

Movie S1 Centrosome migration in a living female interphase GSC. In this movie, one GSC centrosome is localized at the niche-GSC interface, while the other centrosome moves between 10° and 15° around the circumference of the GSC toward the posterior. Figure S2 contains representative frames from this movie. In this movie and in Movie S2, centrosomes are marked by a GFP-tagged pericentrosomal protein Centrosomin (GFP-Cnn, shown in green) under the control of germline-specific Nos-Gal4 driver. The nuclei of the somatic niche cells (and to a lesser extent, the undifferentiated anterior germline cells) are marked by a living-cell DNA dye (Draq5-Cy5, shown in red). The boundary of Cnn-GFP and bright Draq5 staining delineates the CpC-GSC interface. Each movie covers about 30 min of development, with one frame taken every minute. Each frame is a projection of a stack of eight 1 µm confocal slices. The images were taken using a 63× water lens of a DSU Olympus Spinning Disk Confocal Microscope (see more details in Text S1).
(MOV)

Movie S2 Stable positioning of centrosomes in a living female interphase GSC. In this movie, one GSC centrosome is localized at the niche-GSC interface, while the other centrosome is stably positioned 180° around the circumference.
(MOV)

Text S1 Supplemental Material and Methods.
(DOC)

## Abbreviations

BMP: bone morphogenetic protein
Cb: cystoblast
CpC: cap cell
GFP: green fluorescent protein
GSC: germline stem cell
JNK: Jun N-terminal kinase
PBD: P21-binding-domain
pH3: phospho-Histone H3 Ser10
pMad: phosphorylated Mad
