# Supplementary figures and images for: Niche-Associated Activation of Rac Promotes the Asymmetric Division of Drosophila Female Germline Stem Cells
Source: PLoS Biol. 2012 Jul 3;10(7):e1001357. doi: 10.1371/journal.pbio.1001357 (PMC3389017; doi:10.1371/journal.pbio.1001357)

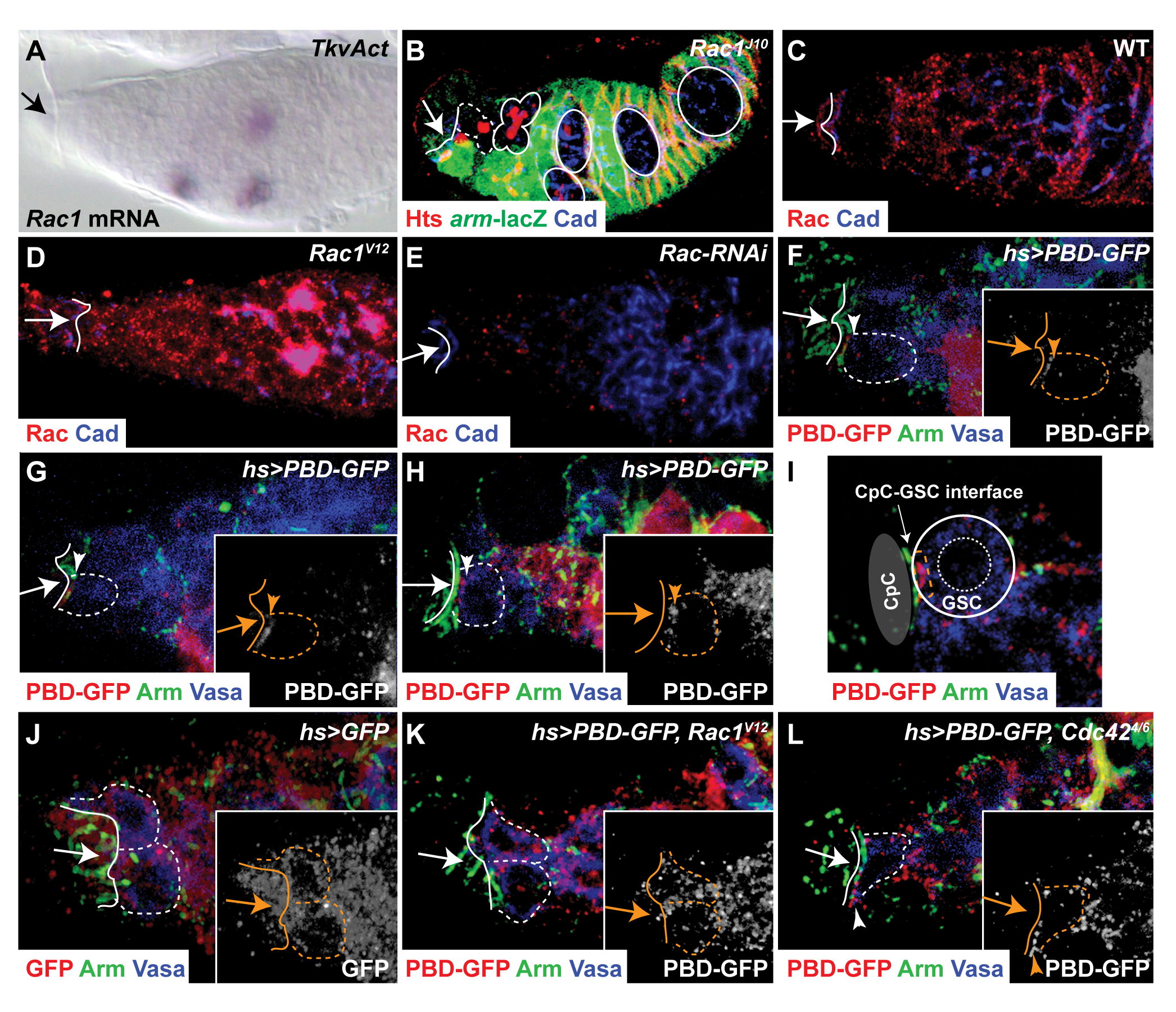

Supplement: Figure S1 — Rac1 is expressed in the germarium and activated at the CpC-GSC interface. (A) A Tkv-Act ovariole hybridized with a Rac1 antisense probe showing Rac1 transcription in three GSC-like cells. (B) A germarium with Rac mutant (Rac1J10 Rac2Δ MtlΔ/Rac1J10 Rac2Δ+) germline clones, marked by absence of lacZ expression, produced by mitotic recombination. Dashed outline, Rac mutant GSC; solid outline, Rac mutant Cb and cyst cells. (C–E) Anti-human Rac1 antibody specifically recognizes Drosophila Rac proteins. Identical staining and image processing of a wild-type ovariole (C), an ovariole from female with germline expression of Rac1V12 (D), and an ovariole from female with germline expression of RNAi against Rac1 and Rac2 (E). (F–H) After mild heat shock, PBD-GFP is localized to the CpC-GSC interface in GSCs. Posterior germ cells were more sensitive to heat shock and usually expressed PBD-GFP uniformly throughout the cytoplasm. (I) Quantitation of the asymmetry of PBD-GFP localization as a ratio of anti-GFP staining at the cortex of the CpC-GSC interface (area enclosed by dashed orange line) to the remainder of the GSC cytoplasm (area enclosed by solid white line) after subtraction of background levels in GSC nucleus (area enclosed by dotted white line). (J–L) PBD-GFP specifically recognizes active Rac in the GSCs. (J) After mild heat shock, GFP alone is localized uniformly throughout the cytoplasm. (K) Mild heat shock was used to drive expression of Rac1V12 and PBD-GFP in the germ line. PBD-GFP is localized to puncta throughout the Rac1V12 GSCs. (L) PBD-GFP is localized to the CpC-GSC interface in females with reduced Cdc42 activity (Cdc424/6). (A–H,J–L) Arrow, CpC niche. (B–H,J–L) Solid line, CpC-GSC interface. (F–H,J–L) Dashed outline, individual GSC. Inset; white, anti-GFP staining. (F–H,L) Arrowhead, asymmetrically localized PBD-GFP in GSCs. (TIF) [file pbio.1001357.s001.tif]

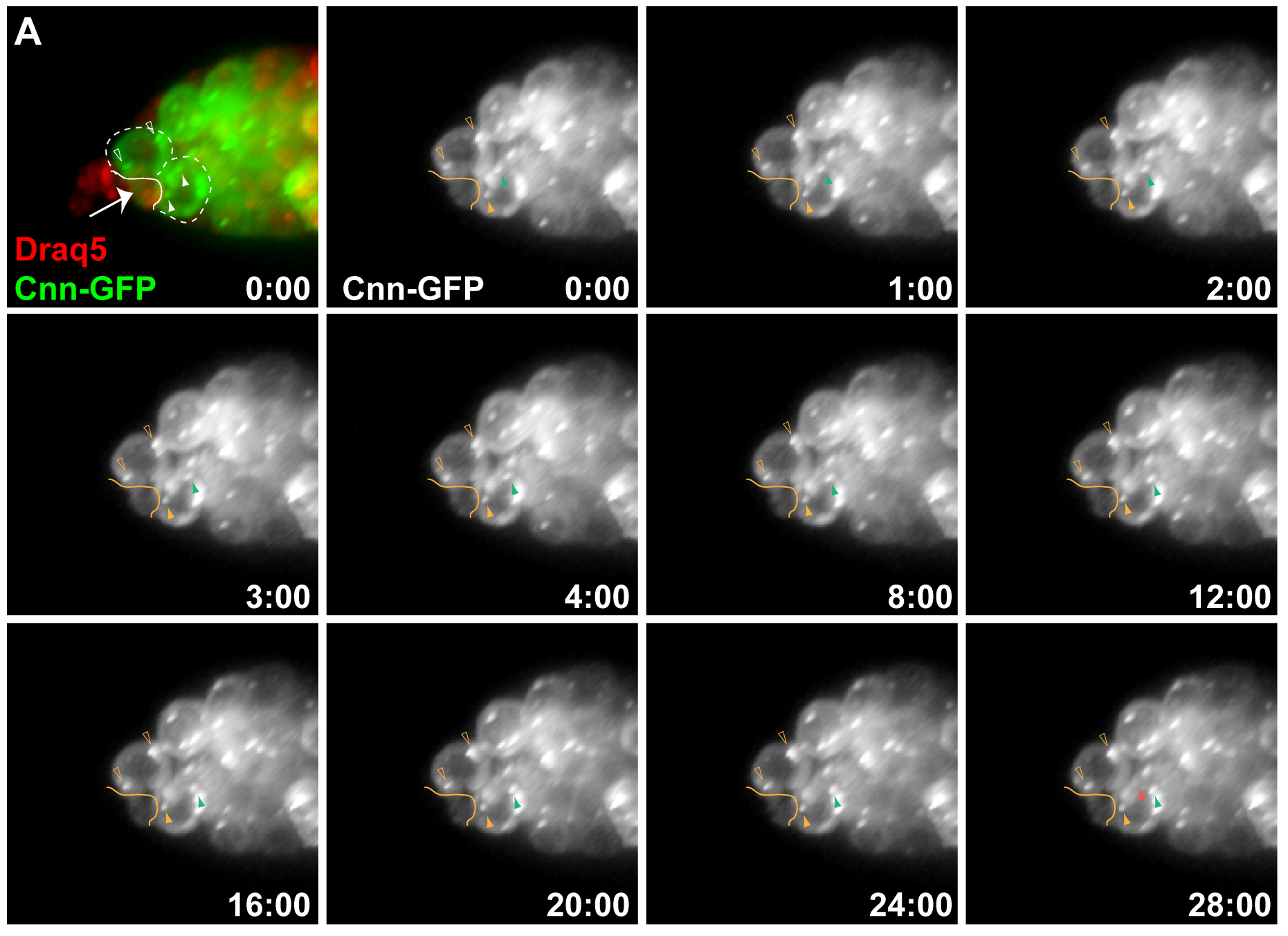

Supplement: Figure S2 — Time series of GSC centrosome migration in a living germarium. (A) Centrosomes, marked by arrowheads, are visualized by a GFP-tagged pericentrosomal protein Centrosomin (GFP-Cnn, green) under the control of germline-specific Nos-Gal4 driver. The nuclei of the somatic niche cells (and to a lesser extent, the undifferentiated anterior germline cells) are marked by a living-cell DNA dye (Draq5-Cy5, red). The boundary of Cnn-GFP and bright Draq5 staining delineates the CpC-GSC interface. The first image displays both channels and demarcates each GSC with dashes; each subsequent image displays only the Cnn-GFP channel. The niche-GSC interface is marked by a solid line and in the first image by an arrow. The series encompasses 28 min; the time of each image is listed. The germarium has two GSCs in focus. The centrosomes in the top GSC (open arrowheads) are positioned perpendicular to the niche GSC interface and do not change position. The centrosomes in the bottom GSC are marked with closed arrowheads: one centrosome is at the niche-GSC interface (closed orange arrowhead); the other is completing its migration around the cortex (closed green arrowhead). In the last image, a closed red arrowhead documents the starting position of the migrating centrosome. (TIF) [file pbio.1001357.s002.tif]

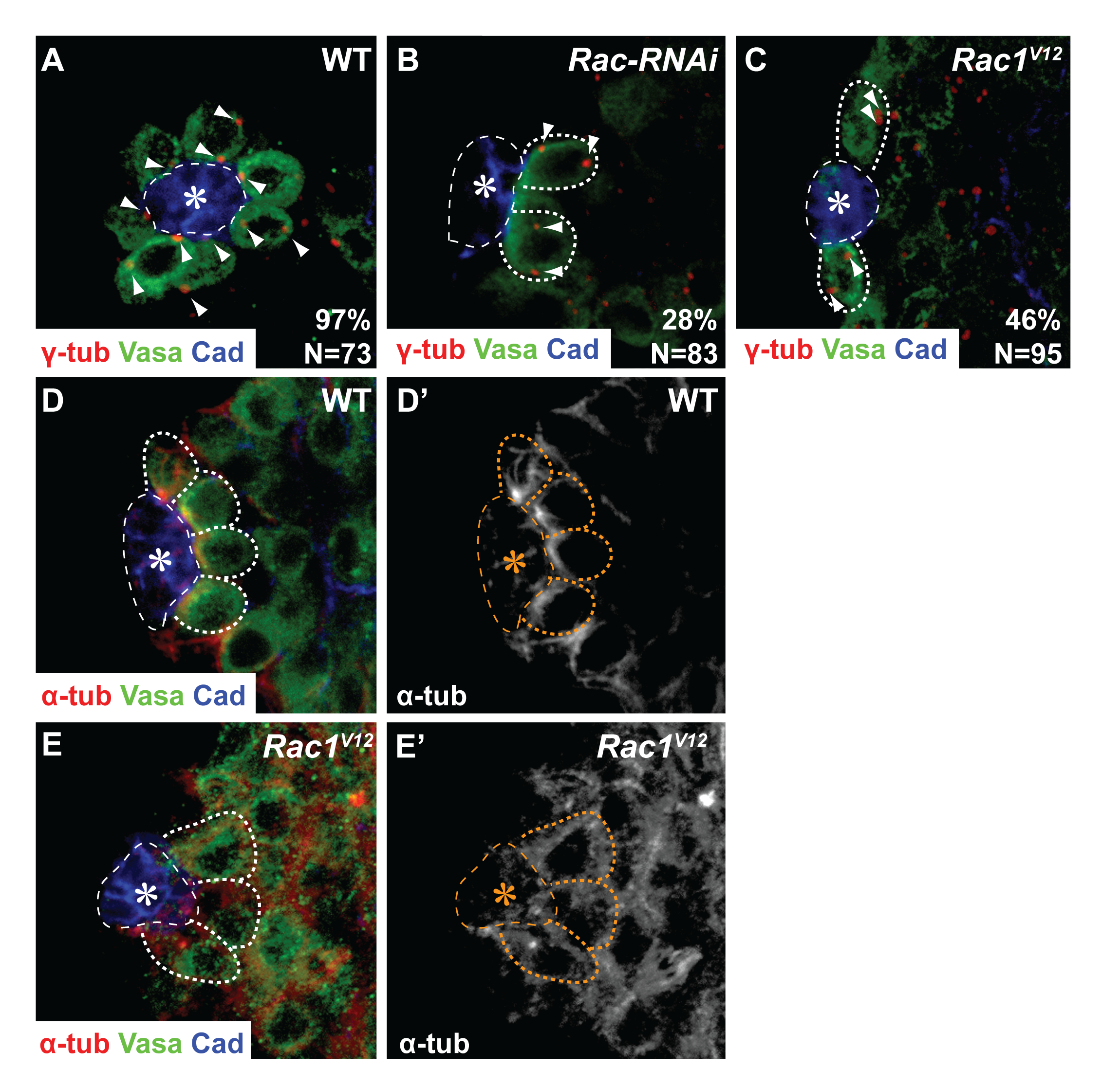

Supplement: Figure S3 — Rac activity controls centrosome position in male GSCs. (A) A male testes with GSCs around their cellular niche, the hub (asterisk). One centrosome in the great majority of wild-type male GSCs is present at the niche-GSC interface. (B–C) Defects in centrosome position in male Rac RNAi (B) or in Rac1V12 (C) GSCs. Two male Rac RNAi GSCs (B) or two male RacV12 GSCs (C) in which neither centrosome is at the niche-GSC interface. (D–D′) In wild-type interphase male GSCs, microtubules are organized as a bundled network near the hub-GSC interface. (E–E′) In Rac1V12-expressing male GSCs, a microtubule network is present uniformly around the GSC cell cortex. (A–E) Anti-Vasa, germline cytoplasm; anti-DE Cadherin, adherens junctions among hub cells and between hub cells and GSCs. (A–C) Anti-γ-tubulin, centrosomes. (D,D′,E,E′) Anti-α-tubulin, cytoplasmic microtubules. (A–E) Asterisk and dashed line, hub cells; dotted outline, individual GSC. (A–C) Percentage (%) of scored (N) GSCs with the phenotype represented in the panel; arrowhead, centrosome. (TIF) [file pbio.1001357.s003.tif]

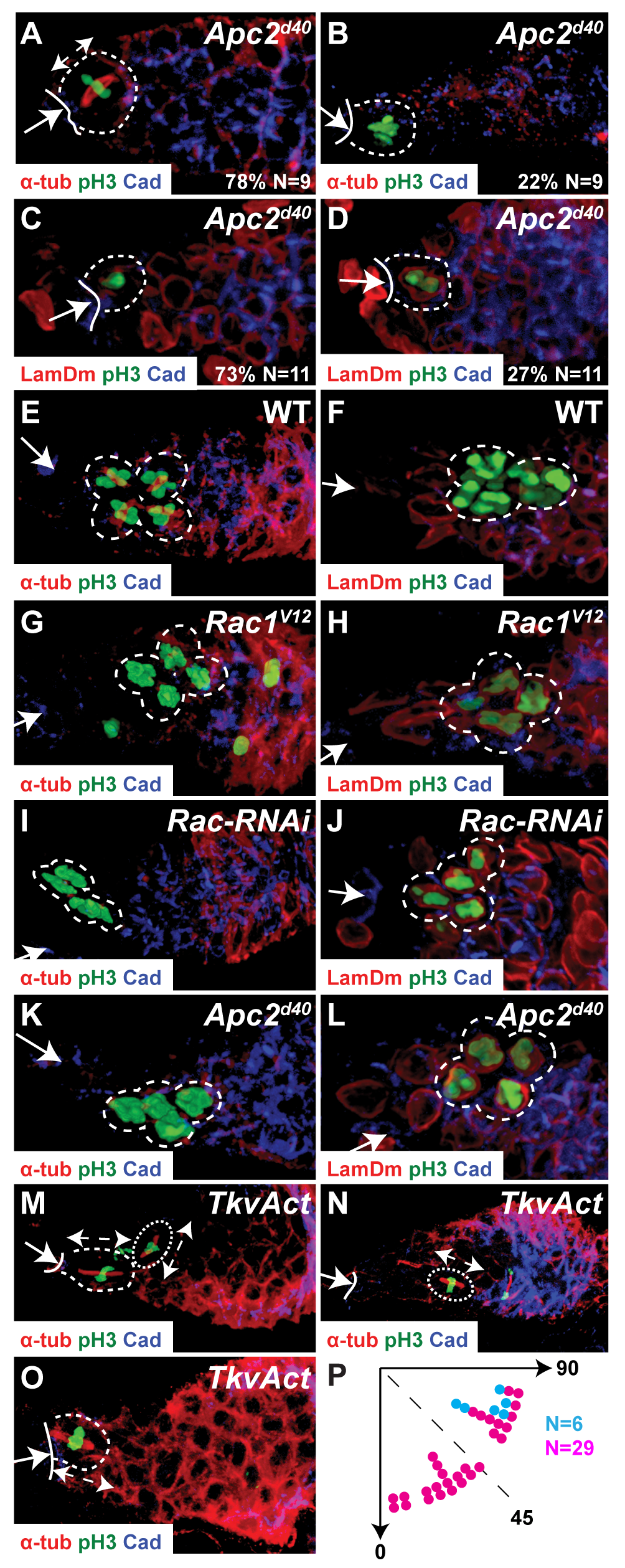

Supplement: Figure S4 — Mitotic phenotypes of Apc2 and TkvAct GSCs and Rac1V12, Rac-RNAi and Apc2 early germline cysts. (A–D) Anti-pH3 stained mitotic Apc2 mutant GSCs. Most pH3 stained Apc2 GSCs have a mitotic spindle with one spindle pole adjacent to the CpC-GSC interface (A) and have undergone nuclear envelope breakdown (C). Some pH3 stained Apc2 GSCs lack a mitotic spindle (B) and have not undergone nuclear envelope breakdown (D). (E–F) Mitotic divisions of wild-type four-cell cysts are synchronous and are marked by condensed chromosomes with mitotic spindle formation (E) and nuclear envelope breakdown (F). (G–L) Mitotic four-cell cysts of Rac1V12 (G,H), Rac-RNAi (I,J), or Apc2 (K,L) with anti-pH3 staining that do not have mitotic spindles (G,I,K) and have not undergone nuclear envelope breakdown (H,J,L). (M–O) Mitotic GSCs and GSC-like cells in TkvAct germaria. While GSCs adjacent to the CpCs niche divide with an invariant division plane (M,O), the division plane of GSC-like cells not at the niche is randomized with respect to the CpC-GSC interface (M,N). (P) Quantification of spindle orientation of dividing niche-residing GSCs and non-niche-associated GSC-like cells in TkvAct germaria. Dot, angle between the CpC-GSC interface and spindle orientation. Cyan dot, dividing GSCs at the CpC niche. Magenta dot, dividing GSC-like cells outside the CpC niche. N, number scored of each of class of GSC. (A–O) Arrow, CpC niche. (A–D,M–O) Solid line, CpC-GSC interface. (A–D) Dashed outline, individual GSC. (E–L) Dashed enclosure, a dividing four-cell cyst. (M–O) Dashed outline, mitotic GSC at the CpC niche; dotted outline, mitotic GSC-like cell outside the niche. (A,M–O) Double-headed arrow, orientation of mitotic spindle. (A–D) Percentage (%) of total (N) GSCs with the specific phenotype displayed in the panel. (TIF) [file pbio.1001357.s004.tif]

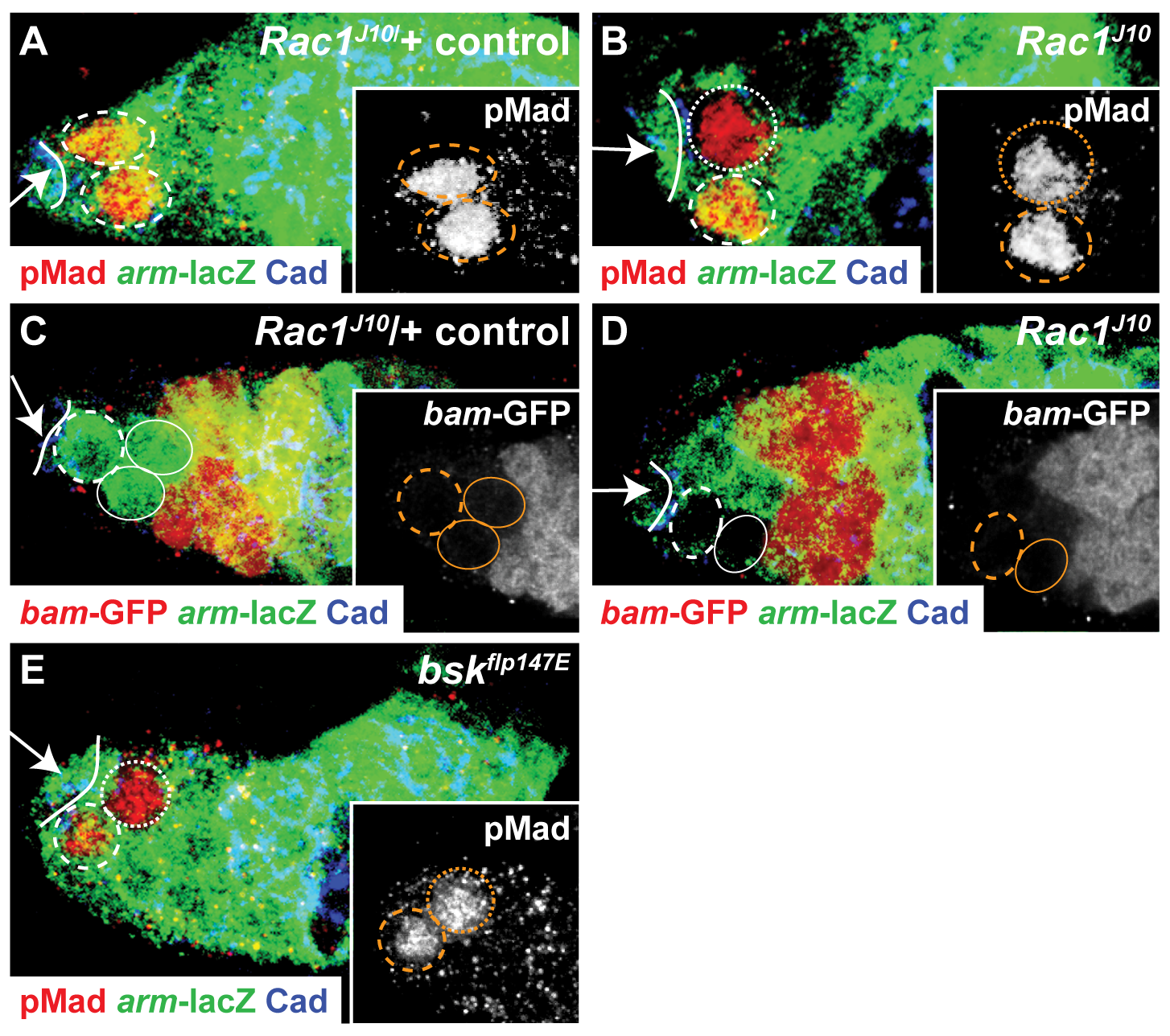

Supplement: Figure S5 — Loss of Rac or JNK activity does not significantly reduce BMP signaling in GSCs. (A) Equivalent levels of anti-pMad staining between GSCs (dashed circles) in a control RacJ10 Rac2Δ MtlΔ/+++ germarium. There was 20.0%±13.6% (n = 8) variability in pMad staining between pairs of GSCs in control germaria. (B) Equivalent levels of anti-pMad staining between one RacJ10 Rac2Δ MtlΔ/+++ GSC (dashed circle) and one RacJ10 Rac2Δ MtlΔ/RacJ10 Rac2Δ+GSC (dotted circle, marked by lack of lacZ expression) after mitotic recombination in a heterozygous female. Homozygous Rac GSCs had on average a 12.8% greater intensity of pMad staining than heterozygous Rac GSCs (n = 17), within the normal range of variability. (C) Lack of bam-GFP expression in a GSC (dashed circle) and extremely low levels of expression in Cbs (solid circles) in a control RacJ10 Rac2Δ MtlΔ/+++ germarium. bam-GFP expression is high in early cysts posterior to Cbs. (D) Lack of bam-GFP expression in a RacJ10 Rac2Δ MtlΔ/RacJ10 Rac2Δ+GSC (dashed circle, marked by lack of lacZ expression) and extremely low expression in a Rac Cb (solid circle, marked by lack of lacZ expression) after mitotic recombination in a heterozygous female. (E) Loss of bsk does not reduce BMP signaling in GSCs. Equivalent levels of anti-pMad staining between a bskflp147E/+ GSC (dashed circle) and a bskflp147E GSC (dotted circle) after mitotic recombination. (A–E) Arrow, CpC niche; solid line, CpC-GSC interface. (A–B,E) Inset: white, anti-pMad staining. (C–D) Inset: white, anti-GFP staining. (TIF) [file pbio.1001357.s005.tif]
