# Supplementary material for: Niche-Associated Activation of Rac Promotes the Asymmetric Division of Drosophila Female Germline Stem Cells
Source: PLoS Biol. 2012 Jul 3;10(7):e1001357. doi: 10.1371/journal.pbio.1001357 (PMC3389017; doi:10.1371/journal.pbio.1001357)
Supplement: Text S1 — Supplemental Material and Methods. (DOC) [file pbio.1001357.s008.doc]

Supporting Materials and Methods – Lu et al.

***Drosophila* strains and culture**

The following RNAi fly stocks are from the National Institute of Genetics (NIG): *P{UAS-Rac1-RNAi*} NIG 2248R-2; *P{UAS-Rac2-RNAi}* NIG 8556R-1. The Kay-GFP CC01156 protein trap line is from the Yale GFP Protein Trap Database. Other fly stocks are as follows: *P{lacZ}Dad^P1883^*/*TM3, Sb* [1] provided by T. Tabata; *P{-198/+133-bam:GFP}* [2] and *P{UASp-GFP}* provided by D. McKearin; *P{A92-lacZ}puc^E69^*/*TM3, Sb* [3], *bsk^flp147E^* *P{neoFRT}40A}*/*CyO* [4], *P{UAS-bsk^.DN^}* (T181A) [5], *P{UAS-Hep^Act^}* (S326D; T330D) [6], P{UAS-*Jun^Asp^.B*} [7], *Cdc42^4^* and *Cdc42^6^* provided by R. Fehon; *P{Gal4::Vp16-nos.UTR}* [8] provided by M. Van Doren; *P{UASp-TkvAct}* [9]; and *dSmurf^15C^* [10].

**Microarray experiments**

After dissection, ovaries were immediately placed in a collection tube in liquid nitrogen and stored frozen at -80º C. Total RNA was purified from TRIzol suspension according to the manufacturer’s instructions (Ambion). The RNA samples were used to synthesize double stranded DNA using a protocol based on the Affymetrix geneChip expression analysis manual (Affymetrix Inc.) and were hybridized to the Affymetrix *Drosophila melanogaster* genome version 1.0 Microarrays using the Affymetrix GeneChip protocol. Three different sets of arrays were used with RNA prepared from flies of the following genotypes: wild-type flies*, yw*; *P{UASp-TkvAct} P{lacZ}Dad^P1883^*/*TM3; TkvAct* flies, *yw; P{Gal4::VP16-nos.UTR}*/*P{UASp-TkvAct} P{lacZ}Dad^P1883^*; *bam* flies *L,cn/CyO; bam^Δ86^*. After the microarray hybridization, RNA and microarray quality assessment controls were performed as suggested by the manufacturer (Affymetrix Inc.). The software “dChip analyzer, 2004” was used to perform statistical analysis of the collected data [11]. The “invariant set normalization method” was used to normalize all arrays at the probe intensity level to a baseline and a “perfect match-only model” was used to compute model-based signal values [11]. The mean intensity for each gene was subject to several criteria to determine if there was a significant difference in the experimental vs. control samples, including at least 1.5 fold or greater change in the expression levels between the means of experimental vs. control samples and an unpaired t-test computed p value of P< 0.05. Using these criteria, 96 genes were found to be differentially expressed between *TkvAct* ovarioles and *bam* mutant ovarioles including genes whose expression was expected to vary between the two samples.

**Molecular Biology**

The *Rac1^V12^* cDNA, including the 5’UTR, was amplified from flies of the following genotype: *yw; P{UAST-Rac1^V12^}*, provided by L. Luo [12], using the following primers: UAST F2, gcgaaagctaagcaaataaaca; UAST R2, gtgcagctttttcctttgtg. The amplified DNA was cloned into the pGEM -T Easy vector (Promega). The *Rac1* cDNA in the pGEM vector was reamplified to introduce *Kpn1* and *Not1* sites using the primers: Kpn1 F1, gtaccacagtccagccgcg gtcacactgcagt; Not1 R1, gcggccgcccgaccaactgtctaactctttt. The resulting PCR product was inserted into the multiple cloning site in the pP{UASp} vector [13] to generate a *P{UASp-Rac1^V12^}* construct. The construct was introduced into flies by standard methodologies.

To generate *P{UASp-PakPBD::eGFP}*, the forward primer PAK-PBD F1 5’- caccatgccgaacatctcctatcc -3’ and the reverse primer PAK-PBD R1 5’- tgtcatgtacttggaacttggcc were used to amplify DNA encoding amino acid residues 81 – 153 of *Drosophila* P21-activated kinase (PAK), encompassing the P21 binding domain (PBD) of the protein, from a cDNA clone (LD20767), obtained from the Drosophila Genome Resource Center. The PCR product was cloned into the pENTR/D-TOPO vector (Invitrogen) and the amplified sequence was verified. The Gateway system (Invitrogen) was used to transfer the insert into the pPWG vector (Terence Murphy, Carnegie Institute of Washington), which allows for expression of C-terminal eGFP tagged proteins under the UASp promoter. The resulting plasmid, *P{UASp-PakPBD::eGFP}*, was sequenced to ensure maintenance of the open reading frame between the PakPBD and the eGFP tag, which are separated by an 18 amino acid residue linker, and was transformed into flies using standard methodologies. A single 3^rd^ chromosome insert, *P{UASp-PakPBD::eGFP}6*, was used in all studies, with one exception: a 2^nd^ chromosome insert *P{UASp-PakPBD::eGFP}4* was used to assay PBD-GFP in *Rac* mutant GSCs.

***in situ* hybridization**

The *in situ* hybridization protocol was described previously [14]. A 2 kb fragment containing most of the coding region of *Rac1* was amplified with the following primers: Rac1F, agccgcggtcacactgc; Rac1R, ttatcttggcttcgttcatgtttt. The resulting PCR product was cloned into pGeM Teasy (Promega). Sense and anti-sense riboprobes were synthesized using T7 and SP6 or T3 RNA polymerases (Roche Applied Science). The riboprobes were labeled with digoxigenin (Roche Applied Science).

**Immunohistochemistry**

Antibodies recognizing the following epitopes were used: γ-tubulin (1:350, mouse, Sigma); α-tubulin (1:1000, mouse, Sigma); phospho-Histone H3 (Ser10) (1:50, rabbit, 06-570 Upstate); DE Cadherin (1:3, rat, Developmental Studies Hybridoma Bank, DSHB); Armadillo (1:5, mouse, DSHB); Hts 1B1 (1:3, mouse, DSHB); Lamin Dm (1:5, mouse, DSHB); Lamin C (1:5, mouse, DSHB); β-galactosidase (1:500, rabbit, Cappel, MP Biomedicals); β-galactosidase (1;500, mouse, Promega); Vasa (1:500, rabbit, provided by P. Lasko); Vasa (1:500, rat, provided by R. Wharton); human Rac1 (1:250, mouse, BD Biosciences Pharmingen; GFP (1:500, rabbit Abcam); PS1 (1:200, rabbit, provided by P. ten Dijke); Apc2 (1:5000, rabbit, provided by M. Bienz). The secondary antibodies used were: Cy3 goat anti-mouse IgG, Cy3 goat anti-rabbit IgG, Cy5 goat anti-rat IgG, Cy5 goat anti-mouse IgG (Jackson ImmunoResearch, all 1:1000); Alexa Fluor 488 goat anti-mouse IgG and goat anti-rabbit IgG (Molecular Probes, Invitrogen, both 1:1000). Prior to use, the anti-pMad and the anti-Apc2 antibodies were pre-absorbed against fixed mixed-stage embryos overnight in an NBT solution at 4°C. Confocal images were obtained with a Zeiss LSM 510 microscope, analyzed using Zeiss LSM Image Browser, prepared using Adobe Photoshop, and assembled by Adobe Illustrator.

**Mitotic recombination to produce *Rac* mutant GSCs**

*Rac* mutant GSC clones were generated according to [15]. Males of genotypes: *P{hsFLP}12*, *y^1^ w^*^*/Y; *Rac1^J11^* *Rac2^Δ^* *P{FRT(w^hs^)} 2A* *Mtl^Δ^*/*TM3*, *Sb* or *P{hsFLP}12*, *y^1^ w^*^*/Y; *Rac1^J10^ Rac2^Δ^* *P{FRT(w^hs^)} 2A* *Mtl^Δ^*/*TM3*, *Sb* or *P{hsFLP}12*, *y^1^ w^*^*/Y; *P{FRT(w^hs^)} 2A* were crossed with *w^*^*; *P{arm-lacZ.V}70C* *P{FRT (w^hs^)}2A* females at 25°C and transferred daily to new bottles. To assay the expression of a transcriptional *bam*-GFP fusion in *Rac* mutant GSCs, males of genotype *P{hsFLP}12*, *y^1^ w^*^/*Y; *P{arm-lacZ.V}70C* *P{FRT (w^hs^)} 2A* were mated to females of genotype *P{-198/+133-bam:GFP}*, *w^*^*; *Rac1^J10^ Rac2^Δ^* *P{FRT(w^hs^)} 2A Mtl^Δ^*/*TM3*, *Sb*. For each experiment, bottles were heat shocked twice on day seven of development for one hour in a 37°C in a water bath, with a one hour interval between heat shocks. Non-TM3 F1 females were collected at 25°C and flipped into new vials with fresh yeast every day. The phenotypes of *Rac* mutant GSCs were assayed by dissecting ovaries from females 7 days after eclosion and staining with antibodies specific for each experiment.

The requirement for *Rac* activity in GSC maintenance was determined by inducing mitotic recombination, dissecting ovarioles from females of different ages after heat shock, staining the ovarioles with anti-β-galactosidase (lack of *lacZ* expression to mark clones), anti-DE-Cadherin (to mark the CpC- GSC interface), and anti-Vasa (to mark germ cells), and using fluorescence microscopy to assay the percentage of germaria of each genotype with marked clones. The initial rate of clone formation was determined by measuring the percentage of germarium with marked clones from females 2-7 days post eclosion. Because each genotype had slightly different initial rates of clone formation (24 – 30%), these values were normalized to 100%. The number of germaria with marked clones from flies at two or three weeks of age were plotted as a percentage of the normalized initial rate. With one exception (n = 245), greater than 350 ovarioles were assayed for each time point for each genotype. Because the assay did not directly measure retention of marked GSCs in the niche, about 100 ovarioles of each genotype with marked clones in the germarium were assayed with confocal microscopy for the presence of a marked GSC. Independent of genotype and age, approximately 60% of the germaria with a marked clone had a marked GSC. Thus, the rate of loss of germarial clones can be taken as a measure of the rate of loss of GSCs from their niche.

Quantitation of BMP signaling in *Rac* homozygous mutant GSCs compared to *Rac* heterozygous GSCs was performed by determining the sum of pMad staining intensity in individual GSCs in stacks of confocal image sections using Image J with the Measure Stack Plugin. The percentage difference in pMad intensity between pairs of GSCs of equivalent genotype was calculated as the difference between intensity of staining in one GSC and the average intensity of staining in both GSCs both divided by the average intensity. The percentage difference in intensity between *Rac* homozygous and *Rac* heterozygous GSCs was calculated as the difference between *Rac* homozygous GSC intensity and *Rac* heterozygous GSC intensity divided by the intensity in the *Rac* heterozygous GSC.

***bsk* mutant GSCs**

To produce GSCs lacking *bsk* activity, males of genotype *P{hsFLP}12*, *y^1^* *w^*^/*Y; *bsk^flp147E^* *P{neoFRT}40A/CyO* were crossed with *w^*^*; *P{arm-lacZ.V}36BC* *P{neoFRT}40A* females at 25°C and transferred daily to new bottles. F1 flies were subject to the same heat shock regimen as described above and ovaries were dissected from females 2-3 days after eclosion.

***Rac-RNAi* knockdown**

Males of genotype *P{UAS-Rac1-RNAi}, w; P{UAS-Rac2-RNAi}* were crossed with *P{Gal4::VP16-nos.UTR}* females at 29°C and transferred daily to new bottles. F1 flies were kept at 29^o^ C with fresh yeast for 7-14 days after eclosion.

**Localization of activated Rac in the GSC**

A heat shock protocol identical to that described in the main Experimental Procedures was used to localize Rac activity in control females of genotypes *P{Gal4-Hsp70.PB}2*/*P{UASp-Rac.V12}*; *P{UASp-PakPBD::eGFP}6*/+ and *P{Gal4-Hsp70.PB}2*/+; *P{UASp-GFP}*/+ and *Cdc42^4^*/*Cdc42*^6^; *P{Gal4-Hsp70.PB}2*/+; *P{UASp-PakPBD::eGFP}6*/+. To localize PBD-GFP in *Rac* mutant GSCs, mitotic recombination in females of genotype *P{hsFLP}12*/+; *P{Gal4-Hsp70.PB}2*/*P{UASp-PakPBD::eGFP}4*; *Rac1^J11^ Rac2^Δ^* *P{FRT(w^hs^)} 2A* *Mtl^Δ^/P{arm-lacZ.V}70C* *P{FRT (w^hs^)}2A* was induced with standard procedures on day seven of development. Females were at maintained at 25^o^ C for seven days after eclosion. After the same heat shock protocol described above, ovaries were dissected and stained with anti-GFP, anti-β-galactosidase (absence of *lacZ* expression marks the *Rac* mutant clones), and anti-DE-Cadherin antibodies.

To quantitate PBD staining in a GSC, a thin (3-5 sections) Z stack was analyzed by ImageJ (NIH) with the Measure Stack Plugin (OptiNav, Inc.) to determine the anti-GFP staining per unit area in different regions of the GSC. The staining at the CpC- GSC interface was determined by calculating the fluorescence intensity per unit area at the cortex of the GSC next to the CpC (as measured by anti-Armadillo staining). The staining in the remainder of the GSC cytoplasm was determined by calculating the fluorescence staining per unit area of the remainder of the GSC cytoplasm (presence of anti-Vasa staining). Prior to calculating the ratio of the two values, a background level of staining, measured as the staining per unit area in the GSC nucleus (lack of anti-Vasa staining), was subtracted from each value. For each cell, the quantification was independently repeated three times and the average of the three ratios was used as the value for that cell.

**Effect of Bam expression on nuclear localization of pMad**

Females of genotypes (1) *yw*; *P{hs-bam.O}18d*/+; *P{Gal4::VP16-nos.UTR}*/*P{UASp-TkvAct}*, (2) *yw*; *P{Gal4::VP16-nos.UTR}*/*P{UASp-TkvAct}*, (3) *yw*, (4) *yw*; *P{hs-bam.O}18d*/+, (5) *yw*; *Smurf^15C^*/*Smurf^15C^*, and (6) *yw*; *Smurf^15C^*/*Smurf^15C^; P{hs-bam.O}18d/+* were heat shocked in a 37° water bath for two hours and returned to room temperature for four hours. The ovaries were then dissected and stained with anti-Arm, anti-pMad, and anti-Vasa antibodies. Vasa is restricted to the cytoplasm of each germ cell, thus delimiting the nucleus. To determine the percentage of *TkvAct* germ cells with nuclear-localized pMad, a Z stack of each germarium was analyzed using the ImageJ program (NIH) with the Cell Counter Plugin (K. De Vos).

**Live imaging of centrosome orientation in GSCs**

Ovaries from the females of genotype *w*, *P{UASp-GFP-Cnn}*/+; *P{Gal4::VP16-nos.UTR}*/+ were dissected in pre-warmed S2 medium (Invitrogen) with 10% IMS (Insect Medium Supplement, Sigma) and Penicillin/Streptomycin (Thermo). The anterior tips of the ovarioles were teased apart and cleaned of muscle sheath using tweezers, then were mixed with S2 medium with 0.5% LMP Agarose and Nuclear Red (Draq5, 1:1000, eBiosciences), and finally were transferred to an image chamber constructed in a 6.5cm Petri dish. After the Agrose-S2 medium solidified, a drop (~200µl) of S2 medium was added to the top of the sample and the Petri dish was covered. The sample was imaged by an Olympus DSU Spinning Disc Confocal (Cy5 and GFP) and analyzed using Slidebook 4.2.

References

1. Tsuneizumi K, Nakayama T, Kamoshida Y, Kornberg TB, Christian JL, et al. (1997) Daughters against dpp modulates dpp organizing activity in Drosophila wing development. Nature 389: 627-631.

2. Chen D, McKearin DM (2003) A discrete transcriptional silencer in the bam gene determines asymmetric division of the Drosophila germline stem cell. Development 130: 1159-1170.

3. Martin-Blanco E, Gampel A, Ring J, Virdee K, Kirov N, et al. (1998) puckered encodes a phosphatase that mediates a feedback loop regulating JNK activity during dorsal closure in Drosophila. Genes Dev 12: 557-570.

4. Riesgo-Escovar JR, Jenni M, Fritz A, Hafen E (1996) The Drosophila Jun-N-terminal kinase is required for cell morphogenesis but not for DJun-dependent cell fate specification in the eye. Genes Dev 10: 2759-2768.

5. Adachi-Yamada T, Nakamura M, Irie K, Tomoyasu Y, Sano Y, et al. (1999) p38 mitogen-activated protein kinase can be involved in transforming growth factor beta superfamily signal transduction in Drosophila wing morphogenesis. Mol Cell Biol 19: 2322-2329.

6. Weber U, Paricio N, Mlodzik M (2000) Jun mediates Frizzled-induced R3/R4 cell fate distinction and planar polarity determination in the Drosophila eye. Development 127: 3619-3629.

7. Treier M, Bohmann D, Mlodzik M (1995) JUN cooperates with the ETS domain protein pointed to induce photoreceptor R7 fate in the Drosophila eye. Cell 83: 753-760.

8. Van Doren M, Williamson AL, Lehmann R (1998) Regulation of zygotic gene expression in Drosophila primordial germ cells. Curr Biol 8: 243-246.

9. Casanueva MO, Ferguson EL (2004) Germline stem cell number in the Drosophila ovary is regulated by redundant mechanisms that control Dpp signaling. Development 131: 1881-1890.

10. Podos SD, Hanson KK, Wang YC, Ferguson EL (2001) The DSmurf ubiquitin-protein ligase restricts BMP signaling spatially and temporally during Drosophila embryogenesis. Dev Cell 1: 567-578.

11. Li C, Wong WH (2001) Model-based analysis of oligonucleotide arrays: expression index computation and outlier detection. Proc Natl Acad Sci U S A 98: 31-36.

12. Luo L, Liao YJ, Jan LY, Jan YN (1994) Distinct morphogenetic functions of similar small GTPases: Drosophila Drac1 is involved in axonal outgrowth and myoblast fusion. Genes Dev 8: 1787-1802.

13. Rorth P (1998) Gal4 in the Drosophila female germline. Mech Dev 78: 113-118.

14. Jiang J, Hoey T, Levine M (1991) Autoregulation of a segmentation gene in Drosophila: combinatorial interaction of the even-skipped homeo box protein with a distal enhancer element. Genes Dev 5: 265-277.

15. Xu T, Rubin GM (1993) Analysis of genetic mosaics in developing and adult Drosophila tissues. Development 117: 1223-1237.
